# Assessment and Optimization of 2T Perovskite/CIGS Tandems via Data-Driven and Optoelectronic Modelling

**DOI:** 10.1007/s40820-026-02141-8

**Published:** 2026-03-31

**Authors:** Gemma Giliberti, Guillermo Farias-Basulto, Klaus Jäger, Thede Mehlhop, Christian A. Kaufmann, Aldo Di Carlo

**Affiliations:** 1 CHOSE–Centre for Hybrid and Organic Solar Energy, Department of Electronic Engineering, Tor Vergata University of Rome, via del Politecnico 1, 00133 Rome, Italy; 2 PvcomB/Helmholtz-Zentrum Berlin für Materialien und Energie GmbH, Schwarzschildstr. 3, 12489 Berlin, Germany; 3 Department Optics for Solar Energy, Helmholtz-Zentrum Berlin für Materialien und Energie GmbH, Albert-Einstein-Str. 16, 12489 Berlin, Germany; 4 Computational Nano Optics, Zuse Institute Berlin, Takustr. 7, 14195 Berlin, Germany; 5 CNR-ISM Consiglio Nazionale delle Ricerche–Istituto di Struttura della Materia, Via del Fosso del Cavaliere 100, 00133 Rome, Italy

**Keywords:** Physics-based modelling, Perovskite/CIGS tandems, Outdoor operation, Perovskite database, Halide perovskite

## Abstract

The global transition to a fossil-free energy system highlights the need for photovoltaic technologies that combine high efficiency with scalability, durability, and flexibility. Monolithic perovskite/Cu(In,Ga)Se_2_ tandem solar cells (TSCs) are an attractive thin-film alternative to perovskite/silicon TSCs, combining low-temperature processing, mechanical flexibility, and radiation tolerance. However, their practical potential remains constrained by coupled optical, electronic, and interfacial losses. This work develops a modelling approach integrating calibrated optical simulations, drift–diffusion device modelling, and a Shockley–Queisser (SQ) formalism, which incorporates empirical non-radiative recombination factors extracted from an external database. Starting from a certified 24.6% Helmholtz-Zentrum Berlin für Materialien und Energie GmbH TSC, the analysis identifies the dominant bottlenecks, quantifies the impact of defect passivation, optical optimization, and bandgap tuning, and extends the assessment to annual energy yield across representative climates. The results estimate practical efficiency limits above 35% and demonstrate significant energy-yield gains under real outdoor conditions, compared with perovskite and Cu(In, Ga)Se_2_ stand-alone devices, for both fixed-tilt and one-axis tracking configurations. At the same time, the database-informed SQ formalism provides a realistic benchmark by linking empirical non-radiative recombination factors to device performance, supporting consistent assessment across tandem architectures. Overall, these findings position perovskite/CIGS tandems as credible high-performance candidates within the landscape of next-generation photovoltaic technologies.

**Supplementary Information:**

The online version contains supplementary material available at 10.1007/s40820-026-02141-8.

## Highlights

- Combined experimental characterization and physics-based modelling of the certified 24.6% perovskite/CIGS tandem reveal CdS/CIGS interfacial recombination and perovskite bulk defects induced by surface roughness.
- Stepwise optimization integrating calibrated simulations with a data-driven Shockley–Queisser analysis demonstrates an efficiency potential exceeding 35% through targeted defect passivation and optical refinements.
- Controlled bandgap tuning further enhances both the achievable efficiency and annual energy yield, provided the material quality is preserved across the full device stack.

**Supplementary Information:**

The online version contains supplementary material available at 10.1007/s40820-026-02141-8.

## Graphical abstract

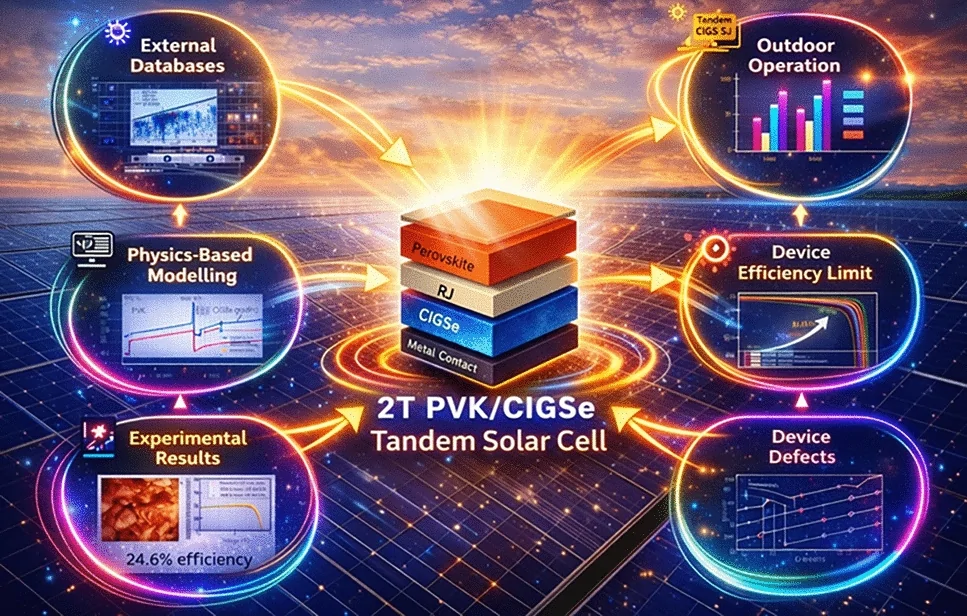

## Introduction

In the twenty-first century, global electricity demand is rising rapidly, driven by industrial growth and the electrification of transport and residential heat [1]. At the same time, the expansion of digital infrastructures, particularly data centres and artificial intelligence platforms, is expected to further increase electricity use, with projections indicating that these sectors alone could account for up to 12% of US demand within the next decade [2, 3]. Meeting this challenge requires the swift decarbonization of power generation to curb greenhouse-gas emissions and places renewable energy technologies at the heart of climate strategies [4, 5]. Among these, photovoltaics (PV) has emerged as the fastest growing and most cost-effective technology, combining scalability, versatility, and minimal environmental impact [6].

Within PV, perovskite (PVK)-based tandem solar cells (TSC) have become one of the most promising routes to surpass the single-junction efficiency limit [7, 8]. Recent records, 34.85% power conversion efficiency (PCE, *η*) for PVK/silicon, 30.1% for all-PVK [9], and 28% for PVK/CIGS TSCs, highlight their competitiveness [10]. While silicon remains the dominant bottom-cell material [11–13], PVK/CIGS tandems provide a full thin-film alternative in which both absorbers can be deposited at low temperature using solution or vacuum processes [14–16].

A key strength of this architecture is the tunability of the absorber bandgaps: wide-bandgap perovskites can be tailored for the top cell, while CIGS offers a continuously adjustable bandgap from 1.0 to 1.7 eV range through Ga/(Ga + In) (GGI) grading. This combination enables precise bandgap matching, with CIGS uniquely capable of reaching and demonstrating very low bandgaps (~ 1.0 eV), which are difficult to achieve with stable perovskite compositions [17], thereby maximizing solar spectrum utilization [18, 19]. The result is a thin-film tandem platform that can be manufactured into lightweight, flexible, and mechanically robust devices, ideally suited for applications where mass, plasticity or durability are critical, including aerospace, vehicle-integrated PV (VIPV), and building-integrated PV (BIPV) [20–22].

Beyond these fabrication advantages, both perovskite and CIGS absorbers show remarkable resilience to high-energy particle irradiation, in contrast to crystalline silicon, making them particularly attractive for space applications [23–25]. In addition, tandem operation provides benefits that extend well beyond Standard Test Conditions (STC): the low-temperature coefficient of the perovskite top cell, combined with the complementary spectral response of the CIGS bottom cell, can deliver superior annual energy output compared to single-junction CIGS, especially in hot or spectrally variable climates [26–28].

Building on these technological and operational advantages, this study focuses on a monolithic PVK/CIGS TSC recently certified at 24.6% PCE by Helmholtz-Zentrum Berlin für Materialien und Energie GmbH (HZB) [29]. Specifically, we address two central questions: (i) which mechanisms currently limit device performance, and (ii) how can targeted, physics-based optimizations unlock further efficiency and energy-yield gains. To this end, we build on earlier modelling work that has demonstrated the value of calibrated frameworks in tandem photovoltaics. In perovskite/silicon tandems, such models have proven essential to disentangle optical and electronic losses and to define realistic efficiency potentials beyond the ideal Shockley–Queisser (SQ) limit [30–32]. For PVK/CIGS tandems, Jošt et al. [33] reported a certified 24.2% efficient monolithic device and identified photocurrent mismatch as a key performance limitation, using optical simulations to derive bandgap- and thickness-dependent guidelines for current matching and to assess efficiency and energy-yield potential. In contrast, Procel et al. [34] analysed a heavily current-mismatched PVK/CIGS tandem solar cell (PCE ~ 10%) using calibrated opto-electrical simulations, demonstrating that energy alignment and recombination at the tunnel recombination junction, together with optical current mismatch, dominate the baseline device performance. Building on previous modelling approaches, we develop a framework that combines multi-level analysis, ranging from physics-based device simulations to a modified Shockley–Queisser limit informed by the MaterialZone database [35] and energy-yield analysis, and apply it to a certified state-of-the-art PVK/CIGS tandem operating close to current-matching conditions.

From a device-physics perspective, this modelling framework couples transfer-matrix or net-radiation methods and ray-tracing optical simulations with drift–diffusion device modelling in Sentaurus TCAD [36]. By incorporating experimentally observed features such as the compositional grading of CIGS (varying GGI ratio) and surface roughness, and calibrating against the reference device, the framework quantitatively reproduces the External Quantum Efficiency (EQE) and the Current–Density–Voltage (J-V) characteristics of both stand-alone cells and the tandem device. Building on this calibrated model, we establish a roadmap that identifies the dominant loss channels and quantifies the benefit of their progressive mitigation; first through physics-based device simulations and then through a modified SQ formalism that incorporates empirical non-radiative recombination factors extracted from the MaterialZone database [35]. This benchmarking step provides a realistic performance ceiling and represents a key novelty of our approach. Finally, the analysis is extended beyond STC through annual energy-yield simulations, where variations in perovskite bandgap and optimized layer thicknesses are explored to derive design rules that balance peak efficiency with stable outdoor operation. A full description of the modelling framework is provided in Supplementary Section S1.

## Device and Modelling

### Device

**Fig. 1:**
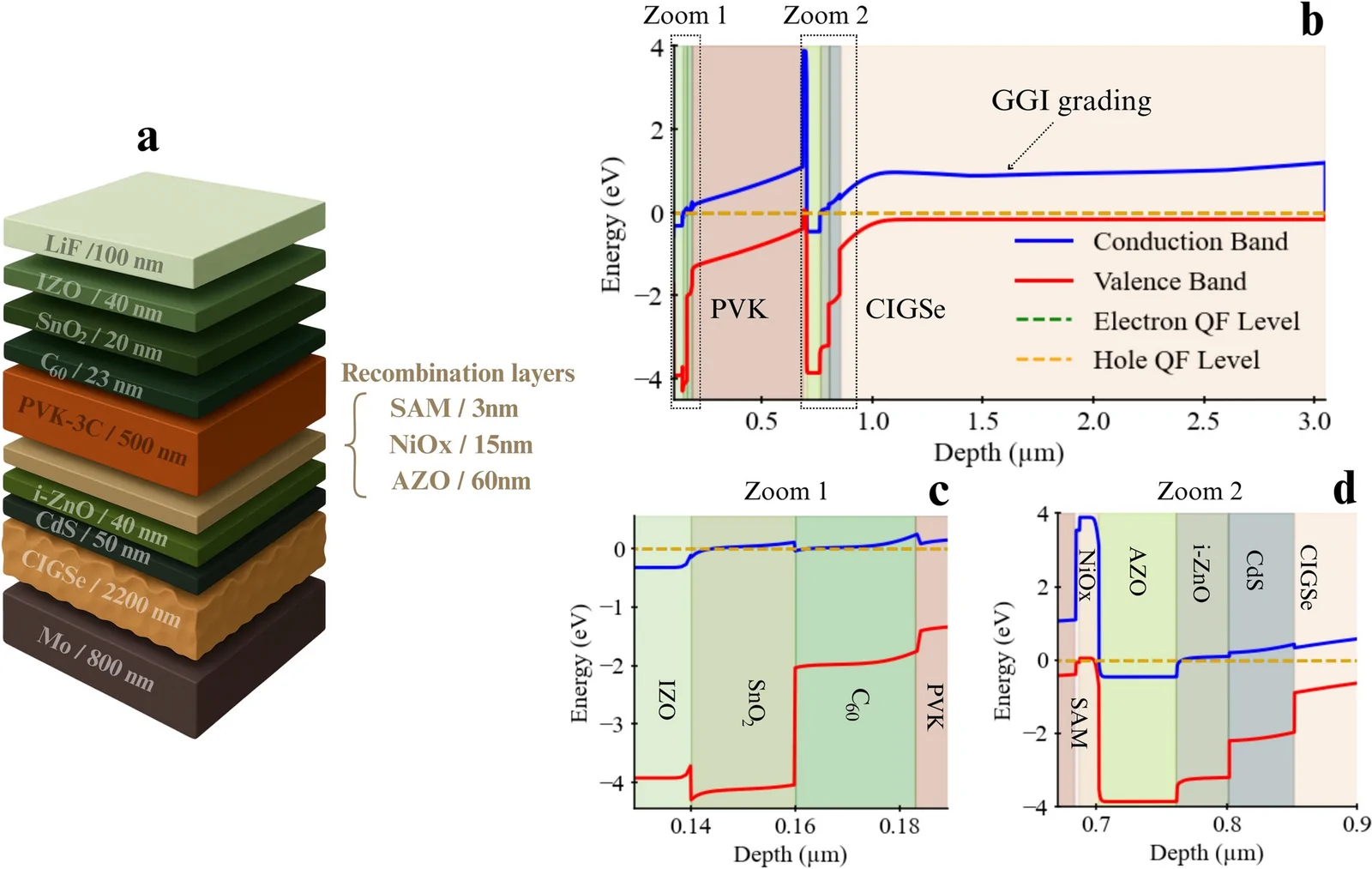
The **a** Layer structure of the monolithic 2-terminal perovskite/CIGS tandem solar cell, comprising a wide-bandgap triple-cation perovskite (PVK-3C, 1.63 eV) top absorber and a graded-bandgap Cu(In,Ga)Se₂ bottom cell. **b** Energy band diagram of the simulated monolithic PVK/CIGS tandem device under equilibrium conditions (in the dark, V = 0), showing the conduction band (blue), valence band (red), and quasi-Fermi levels for electrons (green dashed) and holes (orange dashed). Under these conditions, the electron and hole quasi-Fermi levels coincide and therefore appear as a single Fermi level. **c** Zoomed-in view of the electron-transport-layer (ETL) region, including IZO, SnO_2_, C_60_, and the perovskite absorber. **d** Zoomed-in view of the layers between the PVK and CIGS sub-cells. The complete list of electrical parameters used in the simulations is provided in Tables S2 and S3

Figure 1a illustrates the layer structure of the monolithic perovskite/CIGS tandem solar cell recently certified at 24.6% PCE by HZB [29]. This device lies among the highest certified efficiency values reported for perovskite/CIGS tandems and is therefore representative of the current state of the art, providing a suitable reference for loss analysis and device targeting [9]. Here the quoted thicknesses correspond to the nominal values used in the device simulations. The stack integrates a wide-bandgap (*E*_g_ ≈ 1.63 eV) triple-cation mixed-halide perovskite, Cs_0.05_(FA_0.83_ MA_0.17_)_0.95_Pb(I_0.83_Br_0.17_)_3_ (hereafter PVK-3C or PVK), as the top absorber, with a compositionally graded Cu(In,Ga)Se_2_ bottom cell. The architecture comprises (Fig. 1a): a LiF anti-reflective coating (100 nm); an IZO (40 nm) transparent electrode; a SnO_2_ (20 nm)/C_60_ (23 nm) electron-transport bilayer; the PVK-3C absorber (500 nm); a 2PACz self-assembled monolayer (3 nm); a 15 nm-NiO_x_/Al-doped ZnO (AZO, 60 nm) recombination junction; and the CIGS sub-cell comprising an intrinsic ZnO window (i-ZnO, 40 nm), a CdS buffer (50 nm), a graded CIGS absorber (2.2 µm), and a molybdenum back contact (800 nm). The Ga/(Ga + In) ratio in the CIGS increases from ~ 0.30 at the front to ~ 0.65 at the rear, resulting in a bandgap widening from ~ 1.05 to ~ 1.41 eV (Fig. S2a). This composition profile affects both optical absorption in the CIGS sub-cell and its electrical behaviour. Atomic force microscopy (AFM) reveals a root-mean-square roughness of ~ 147 nm (Fig. S2b), which strongly influences both light scattering and interface quality. Additional experimental details are provided in Section S1.4.

### Modelling

Based on the device structure described above, optical and electrical modelling parameters are assessed to reproduce the device behaviour. Optical inputs, including the Ga/(Ga + In) profile, surface roughness, and the optical constants *n*(λ) and *k*(λ) of the device layers, are provided in Sections S1.1–S1.2, while electrical parameters governing doping, transport, and recombination, consistent with the deposition conditions of the reference cell (Section S1.4), are summarized in Tables S2 and S3 (Section S1.3).

Figure 1b–d shows the simulated energy band diagram obtained with Sentaurus TCAD, under dark conditions [36]. The perovskite sub-cell is modelled in a *p–i–n* configuration, while the CIGSe exhibits the typical curvature of the conduction and valence bands (CB and VB, respectively) induced by Ga grading. In the electron-transport region (Fig. 1c), the conduction-band minimum of IZO lies slightly below that of SnO_2_ (see Table S2), creating a favourable alignment for electron extraction. IZO is treated as a highly doped transparent conductive oxide [37–39], while SnO_2_ parameters are calibrated from ALD-deposited films at 80 °C [40–42]. Both the SnO_2_/C_60_ and C_60_/perovskite interfaces exhibit downward band bending towards the anode, facilitating electron accumulation and extraction [41].

On the hole-transport side (Fig. 1d), a 3-nm-thick 2PACz self-assembled monolayer at the perovskite/NiO_x_ interface improves energy-level alignment [42]. The subsequent NiOₓ/AZO heterojunction (Fig. 1d) exhibits pronounced band bending, induced by the high *p*⁺ doping of NiO_x_ and the high *n*⁺ doping of AZO (see Table S2 for electrical parameters). This results in a narrow depletion region that enables efficient inter-band tunnelling and carrier recombination between the sub-cells [34, 43]. Consequently, the NiOₓ/AZO stack forms the HTL/TCO recombination junction [43], which is modelled in TCAD Sentaurus through a direct band-to-band (B2BT) tunnelling mechanism [34, 36] consistent with the favourable energy-level alignment of the two materials. This configuration represents a further advancement with respect to the PEDOT:PSS (or NiO_x_)/SnO_2_ recombination junction previously discussed by Procel et al. [34].

With the device structure and its materials parameters established, the behaviour of the tandem device is reproduced using a calibrated multiscale simulation framework that integrates optical, electrical, and, when required, circuit-level modelling into a unified scheme (Section S1).

On the optical side, simulations are performed in GenPro4 [44] using the net-radiation method combined with ray tracing to account for light scattering induced by the measured CIGSe roughness. The graded CIGSe absorber is described directly from the experimentally determined Ga/(Ga + In) depth profile (Fig. S2a). Its depth-dependent optical constants are obtained through a custom interpolation routine developed in this work (Section S1.2), which extends the bandgap-shifting approach of Hörantner and Snaith [27] to CIGSe. In this method, reference extinction-coefficient spectra are spectrally shifted to match the local bandgap (ranging from ~ 1.05 eV at the front to ~ 1.41 eV at the back), while the corresponding refractive index is derived via Kramers–Kronig transformation using the open-source package pykk.py library. This procedure yields a continuous and physically consistent set of *n*(λ) and *k*(λ) values across the graded absorber. The resulting wavelength- and depth-resolved generation profiles are then used as input for Poisson–drift–diffusion simulations in Sentaurus TCAD [36], providing a calibrated description of carrier transport and recombination dynamics. For the small-area reference device considered here (active area ~ 1 cm^2^), front-contact losses associated with the metal grid are not explicitly modelled, as a previously optimized grid design is employed [29, 45] (see Fig. S7) and its impact on the overall device performance will show up in scaled-up cells and mainly in the fill factor. The device modelling therefore focuses on intrinsic optoelectronic loss mechanisms.

## Results and Discussion

### Loss Analysis and Bottleneck Identification

**Fig. 2:**
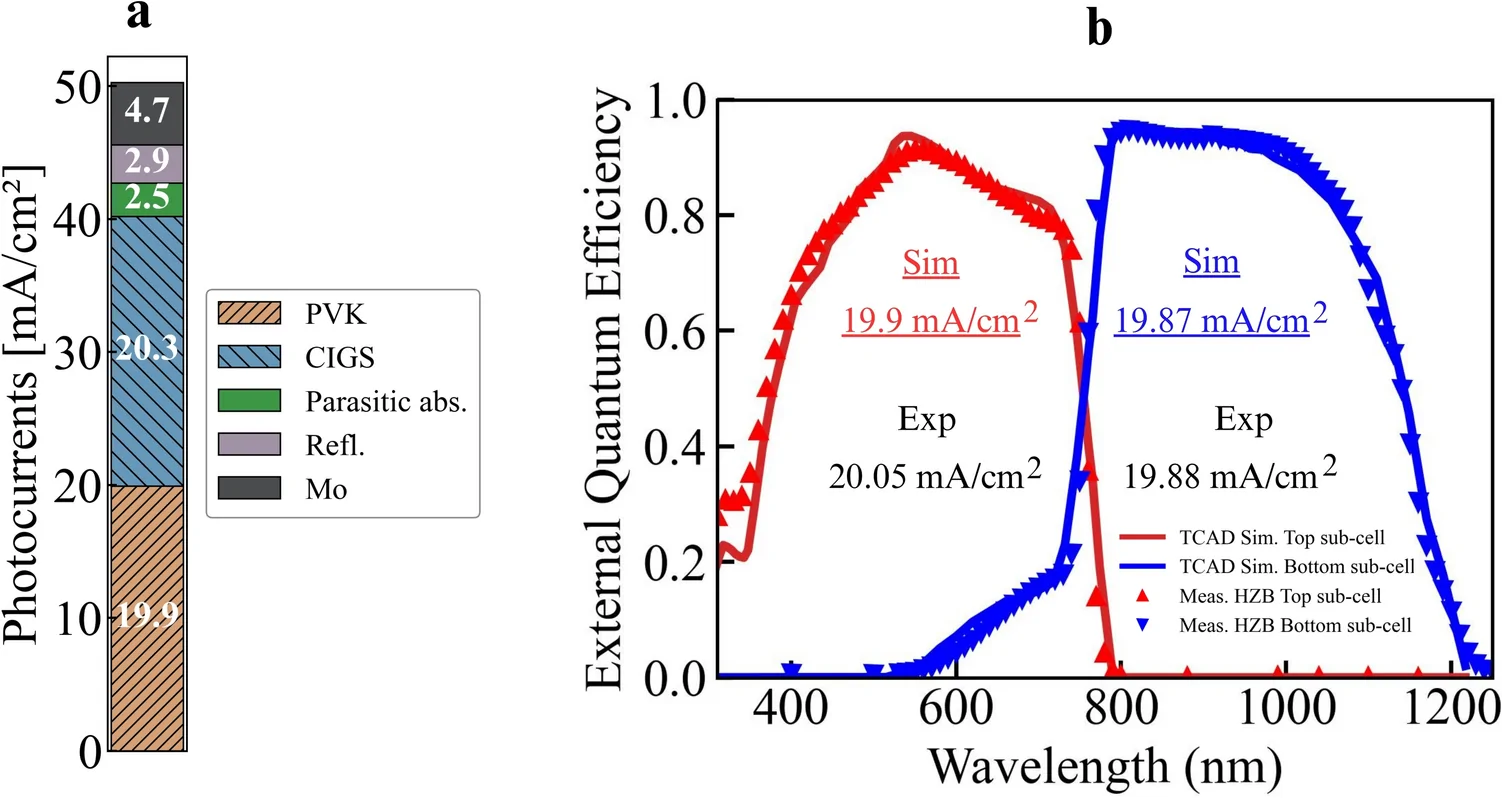
**a** From optical simulation: stacked bar plot showing the simulated photocurrent contributions from each component of the tandem device under AM1.5G illumination. **b** From optoelectronic simulation: External Quantum Efficiency (EQE) of the perovskite (top) and CIGS (bottom) sub-cells in tandem configuration (dotted lines from HZB measurements)

Figure 2a shows the simulated photocurrent contributions under AM1.5G illumination. Optical losses originate primarily from parasitic absorption in non-active layers, which account for more than 4% of the incident photon flux, and from front-side reflection, responsible for a further 5.8%. Accounting for the measured CIGS surface roughness (Fig. S2b), enhanced light trapping in the bottom sub-cell contributes ~ 1 mA cm^−2^ to the simulated CIGS photocurrent, while its influence on the perovskite top-cell current remains negligible. As a result, the PVK and CIGS sub-cells generate 19.9 and 20.3 mA cm^−2^, respectively. A fraction of the CIGS photocurrent originates from photons transmitted through the perovskite at wavelengths close to its absorption edge. Yet, while optical modelling predicts a CIGS photocurrent above 20.3 mA cm^−2^, EQE measurements give a lower value of 19.9 mA cm^−2^ (Fig. 2b). This small discrepancy reflects a combination of residual optical uncertainties near the perovskite absorption edge and recombination losses at the CdS/CIGS interface, which limit carrier collection in the CIGS bottom cell (parameters reported in Table S3). Under operating conditions, the CIGS junction therefore emerges as the current limiting element of the device rather than the perovskite.

**Fig. 3:**
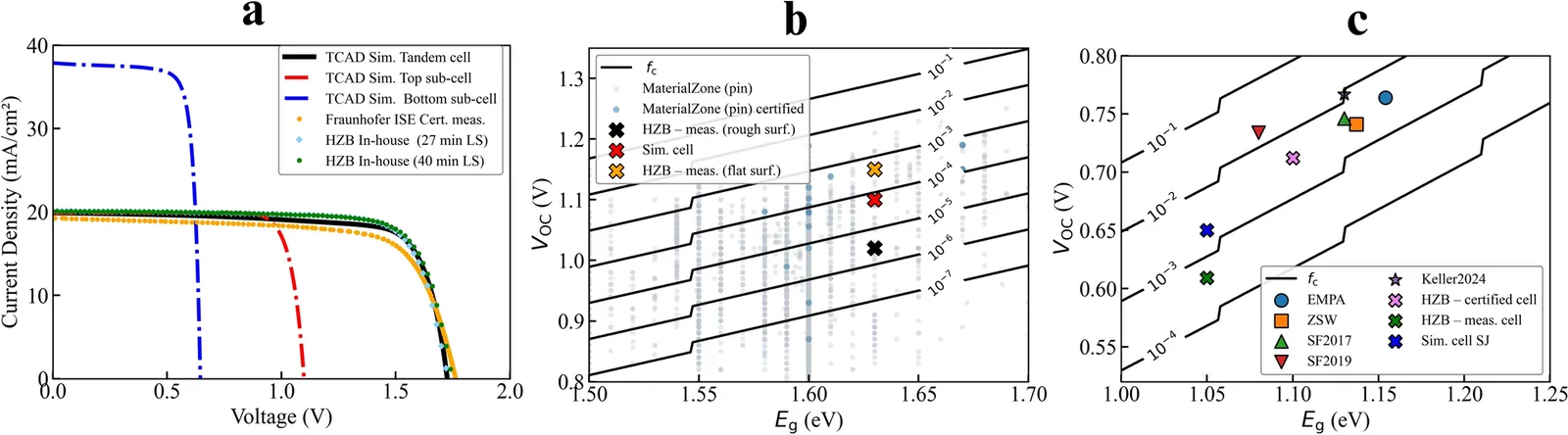
**a** *J-V* curves of stand-alone perovskite (red), CIGS (blue), and tandem (black) simulated devices; dotted lines show certified and in-house data (area = 1.10 cm^2^). *V*_oc_ versus bandgap (*E*_*g*_) for **b** perovskite and **c** CIGS sub-cells, with iso-lines of non-radiative recombination factor (*f*_*c*_)

To corroborate these findings, in Fig. 3a we compare the *J-V* characteristics of the best-performing in-house (*η* ~ 26.8%/27.0% after 27/40 min light soaking), the certified reference (*η* ~ 24.6%) [29], the simulated tandem, and the corresponding stand-alone sub-cells. Because TSC efficiency is ultimately determined by the behaviour of the individual junctions, we first examined the stand-alone devices and benchmarked them against literature data. In this regard, the analysis was extended by plotting the open-circuit voltage (*V*_oc_) as a function of bandgap (Fig. 3b, c) and comparing with reported perovskite data from the MaterialZone database [35] and CIGS trends [46]. For clarity, iso-lines of the non-radiative recombination factor (*f*_*c*_) were added [47]. This parameter, originally introduced within the Shockley-Queisser framework [47], quantifies the ratio of radiative to total recombination. It therefore provides a direct measure of the deviation from the radiative limit (*f*_*c*_ = 1), where all recombination processes are radiative. Plotting iso-lines of *f*_*c*_ helps visualize the voltage loss relative to the measured *V*_oc_ and identify the junction that predominantly constrains the tandem performance (see Section S1.5 for details).

Stand-alone perovskite top cells (i.e. single junction) in both flat and rough surfaces reveal a strong dependence of the *V*_oc_ on substrate morphology (Fig. 3b). Devices fabricated on a flat surface Mo/ITO/glass stack, (orange marker in Fig. 3b), reached 1.15 V after 5 min of light soaking (LS), whereas those deposited on a rough surface Mo/CIGSe/glass stack replicating the tandem’s bottom-cell morphology (see cross-sectional SEM image in Section S1.4), exhibited a markedly lower initial *V*_oc_ of 0.88 V, recovering only partially to 1.03 V after 9 min of LS (black marker in Fig. 3b).

The ~ 120 mV deficit is attributed to the pronounced CIGS roughness, which disrupts uniform perovskite crystallization, increases interfacial defect density, and enhances non-radiative recombination, consistent with previous reports [48, 49]. Accordingly, in Fig. 3b the flat-substrate device approaches the *f*_*c*_ ~ 10^–3^ contour, whereas roughness-induced degradation shifts the *V*_oc_ closer to *f*_*c*_ ~ 10^–6^, indicating a substantial departure from the radiative limit due to interface-related non-radiative losses. This establishes a direct link between the CIGS surface roughness and the observed *V*_oc_ and FF losses in the perovskite sub-cell and, consequently, the performance of the monolithic tandem.

The CIGS bottom cell, in stand-alone configuration, also exhibits non-negligible voltage penalties. Stand-alone AZO/ZnO/CdS/CIGS devices (with *E*_*g*_ = 1.05 eV) (experimental results in Section S1.4) show *V*_oc_ values around 0.60 V, approximately 100 mV lower than the certified HZB CIGS solar cells with 1.1 eV bandgap [50, 51].

When integrated in the monolithic tandem solar cells, this translates into an additional *V*_oc_ penalty of roughly 20 mV. In Fig. 3c, the measured CIGS device lies close to the *f*_c_ ≈ 10^–3^ contour, signifying that non-radiative recombination dominates, however performance remains relatively close to the best reported for comparable bandgaps.

Building on these optical and electrical insights, we tuned the electrical parameters of the simulated stand-alone sub-cells to reproduce the tandem *J–V* response. Defect densities (Table S3) were assigned in accordance with the main recombination mechanisms: bulk and interfacial defects in the perovskite (PVK/LiF/C_60_ and PVK/SAM interfaces), and interface defects at the CdS/CIGS junction in the bottom cell. The chosen values represent a compromise between the measured stand-alone performance and the conditions required to reproduce the behaviour of the integrated tandem. The photovoltaic parameters of all simulated and measured devices are summarized in Table 1.

**Table 1:** Photovoltaic parameters of simulated and experimental device [29]

Devices	*J*_SC_ (mA cm^−2^)	*V*_OC_ (V)	*P*_mpp_ (mW)	FF (%)	PCE (%)
Stand-alone PVK cell sim	19.90	1.10	19.9	82.3	18.0
Stand-alone CIGS cell sim	37.85	0.65	21.7	80.4	19.6
Tandem sim	19.87	1.73	29.0	76.7	26.3
In-house 27 min LS	20.08	1.73	29.6	77.3	26.8
In-house 40 min LS	20.08	1.75	29.8	77.0	27.0
Fraunhofer ISE Cert (mean)	19.28	1.76	26.8	71.3	24.2
Fraunhofer ISE (Steady state record)	–	–	27.2	–	24.6

### Practical Efficiency Potential of Perovskite/CIGS Tandem Devices

**Fig. 4:**
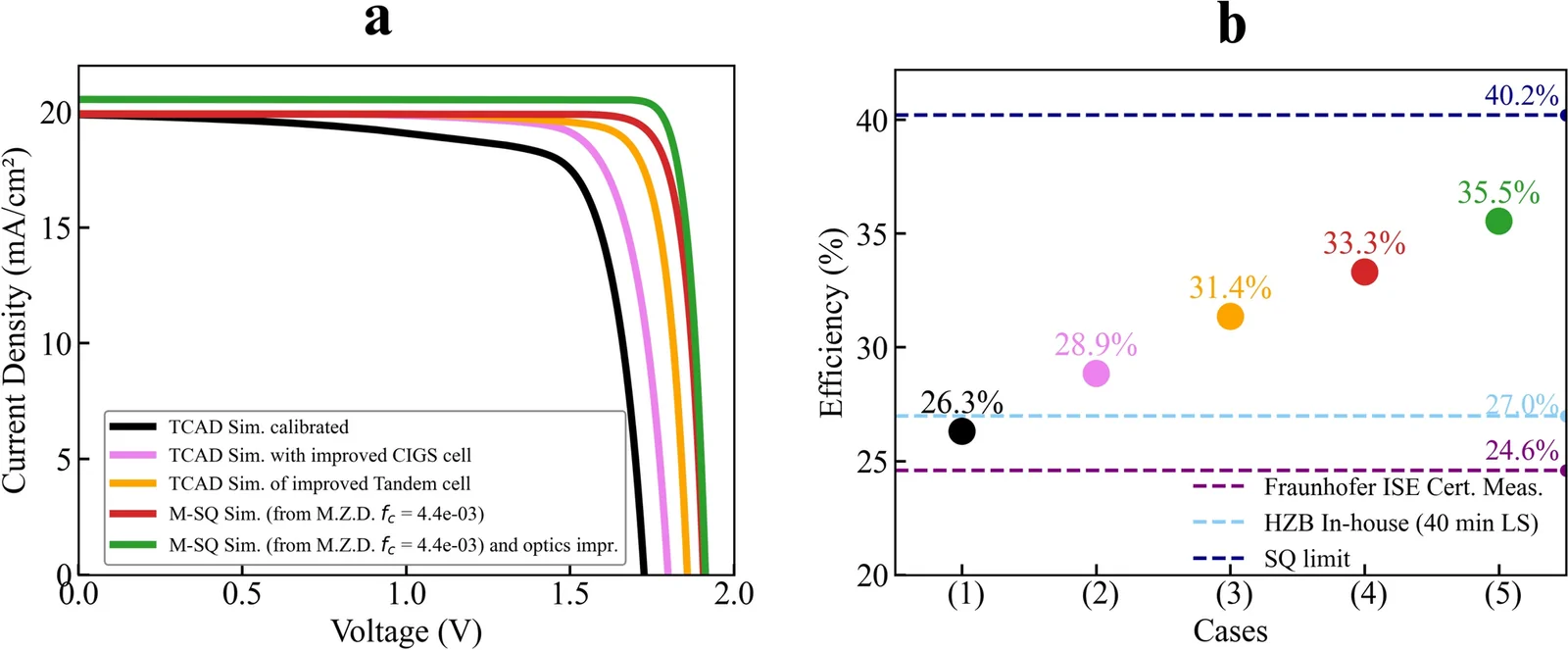
**a** Simulated *J*–*V* curves of the monolithic perovskite/CIGS tandem cell under different scenarios. **b** Corresponding efficiencies for baseline and improved cases, compared with certified and in-house measurements as well as the SQ limit

Using the calibrated optoelectronic model, we analysed a stepwise loss-mitigation pathway to quantify efficiency gains from progressive defect reduction and targeted technological improvements, shown in Fig. 4. Starting from the experimentally validated baseline (Case 1, *η* = 26.3%), the next two steps focus on mitigating defect-related losses. In Case 2, passivation of interface states at the CdS/CIGSe junction (Fig. 1d), recognized as a dominant recombination site in CIGS absorbers [21, 34, 52], raises the bottom-cell *V*_oc_ towards that of the certified stand-alone device, consistent with recent monolithic tandem reports showing gains of ~ 50–60 mV [21]. As a result, the efficiency thereby increases to 28.9%. From a device-physics standpoint, such a *V*_oc_ recovery can be practically targeted via alkali post-deposition treatments and controlled Ga grading near the CdS/CIGSe junction to passivate non-radiative recombination pathways and optimize the conduction-band offset, while ensuring a uniform, low-defect buffer/absorber interface with limited parasitic absorption [53].

In Case 3, mitigating roughness-induced bulk defects in the perovskite sub-cell [10] allows the perovskite *V*_oc_ to approach values measured on flat substrates, further boosting efficiency to 31.4%. Consistent with these observations, previous studies have shown that controlling CIGSe surface morphology is crucial to achieve uniform perovskite crystallization, suppress interfacial defects, and reduce non-radiative recombination [21, 48, 54]. Notably, recent studies show that engineering a controlled corrugated CIGSe interconnection, i.e. a continuous concave–convex morphology can mitigate tensile stress in the perovskite, thereby suppressing non-radiative recombination and enhancing operational stability, while also delivering a modest *V*_oc_ gain [10]. As shown in Fig. 4a, Cases 2 and 3 progressively improve *V*_oc_ and *FF* while keeping the short circuit current density (*J*_sc_) nearly constant (Table 2), shifting the current-limiting sub-cell from CIGSe (Case 1) to the perovskite (Case 2).

**Table 2:** Photovoltaic parameters of simulated devices

Cases	*J*_SC_ (mA cm^−2^)	*V*_OC_ (V)	*J*_mpp_ (mA cm^−2^)	*V*_mpp_ (V)	FF (%)	PCE (%)
(1)	19.87	1.73	17.7	1.5	76.6	26.3
(2)	19.9	1.79	18.8	1.5	80.6	28.9
(3)	19.9	1.86	18.9	1.7	84.7	31.4
(4)	19.9	1.91	19.3	1.7	87.8	33.3
(5)	20.5	1.91	20.2	1.8	90.5	35.5

Beyond defect mitigation, Case 4 projects performance towards state-of-the-art levels by adopting non-radiative recombination factor *f*_c_ representative of the best single-junction devices in the MaterialZone database [35], within a Modified Shockley–Queisser (M-SQ) framework (details in Section S1.5). This provides a conservative yet realistic benchmark (33.3% efficiency), as comparable *f*_*c*_ values have been achieved through interface engineering strategies. For example, on the hole-transport side, NiO_x_-based hole-transport layers can be improved with Cu doping or surface treatments to improve band alignment and charge extraction, thereby enhancing both *V*_oc_ and *FF* [21, 55]. On the electron-transport side, further improvements may be envisioned by substituting conventional ALD-deposited SnO_x_ with a fully physical, “dry” interlayer such as Sn-doped In_2_O_3_ deposited by evaporation. By tuning the Sn-doping content in the interlayer, an improved energy-band alignment between the ETL and the IZO electrode can be achieved [38].

Case 5 finally incorporates ideal optical conditions by eliminating front-surface reflection and parasitic absorption, allowing the photocurrent to approach the Shockley–Queisser limit for the chosen bandgaps. Combined with the voltage improvements of Case 4, this raises the projected tandem efficiency to 35.5%. From an optical standpoint, a minimal yet effective improvement for our device would be a reduction in the C_60_ thickness, which constitutes a non-negligible source of parasitic absorption (1.3 mA cm^−2^) and could therefore help the device approach this efficiency limit.

Figure 4b illustrates that sequential interface passivation and morphological control can elevate efficiency from ~ 26% to above 31%, and that with state-of-the-art material quality and optical optimization, values beyond 35% are realistically attainable under AM1.5G illumination.

**Fig. 5:**
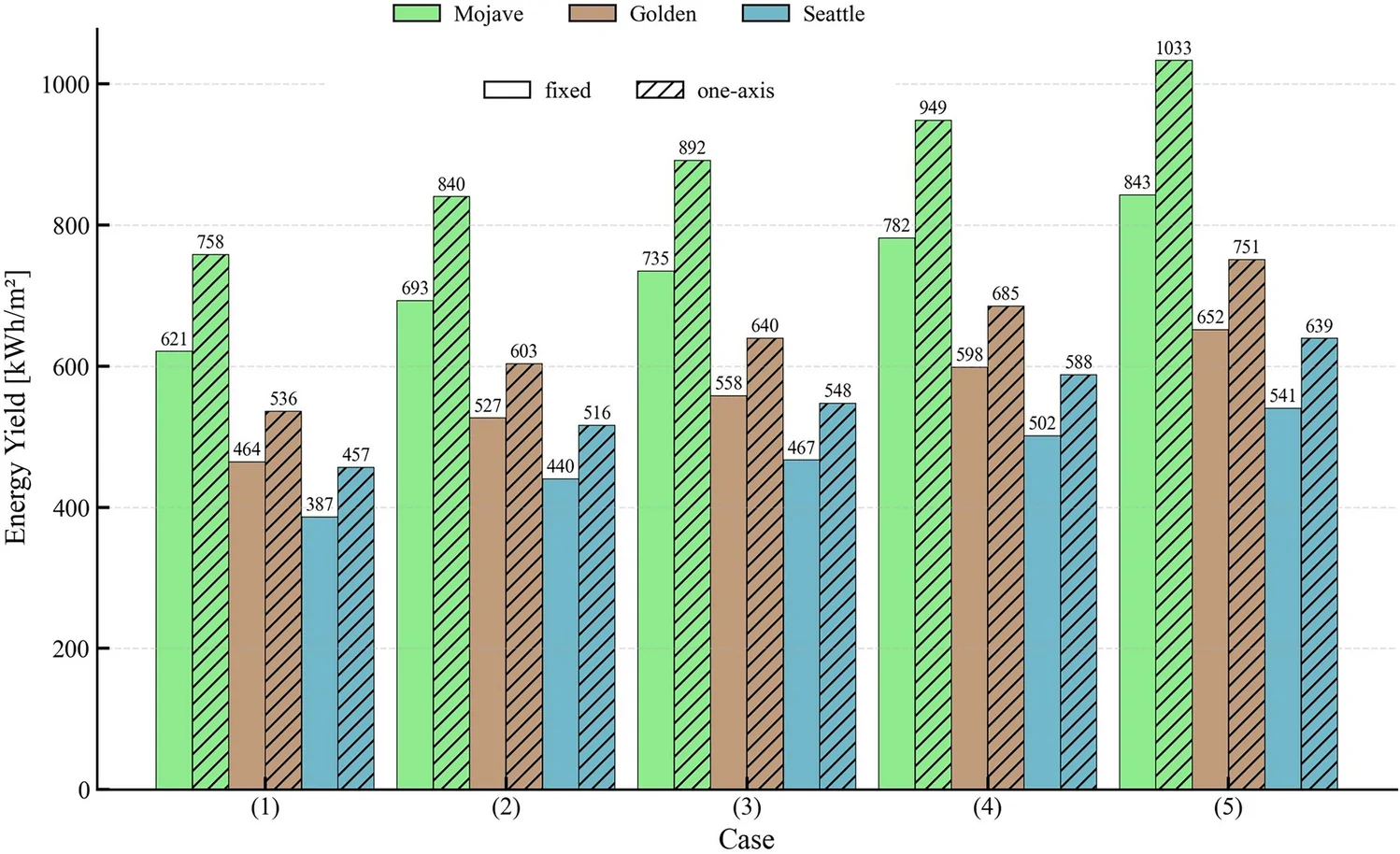
Annual energy yield (EY, kWh m^−2^) for the five step-cases in Mojave, Golden, and Seattle, under fixed-tilt and one-axis tracking configurations

Annual energy-yield (EY) simulations confirm these trends under realistic spectral and thermal conditions (Fig. 5). The calculations were performed using the multiscale simulation workflow described in Fig. S1 and employ typical meteorological year (TMY) spectral data from NREL [56]. Three representative climates were analysed, Mojave (desert, high irradiance), Golden (temperate), and Seattle (diffuse-light dominated), under both fixed and one-axis tracking configurations, consistent with established benchmarks [27].

Across all locations, the cumulative improvements from Case 1 to Case 5 result in a steady increase in energy yield (EY, kWh m^−2^) (Fig. 5). Relative gains are most pronounced in low-irradiance or spectrally variable climates, where enhanced optical management (Case 5) maximizes photon harvesting. One-axis tracking provides an additional 8%–14% boost, further amplifying the benefits of the mitigation steps. These EY gains closely align with previous modelling of perovskite/CIGS tandems [26, 33], which highlighted the need to co-optimize *J*_sc_ and *V*_oc_ for robust outdoor operation.

**Fig. 6:**
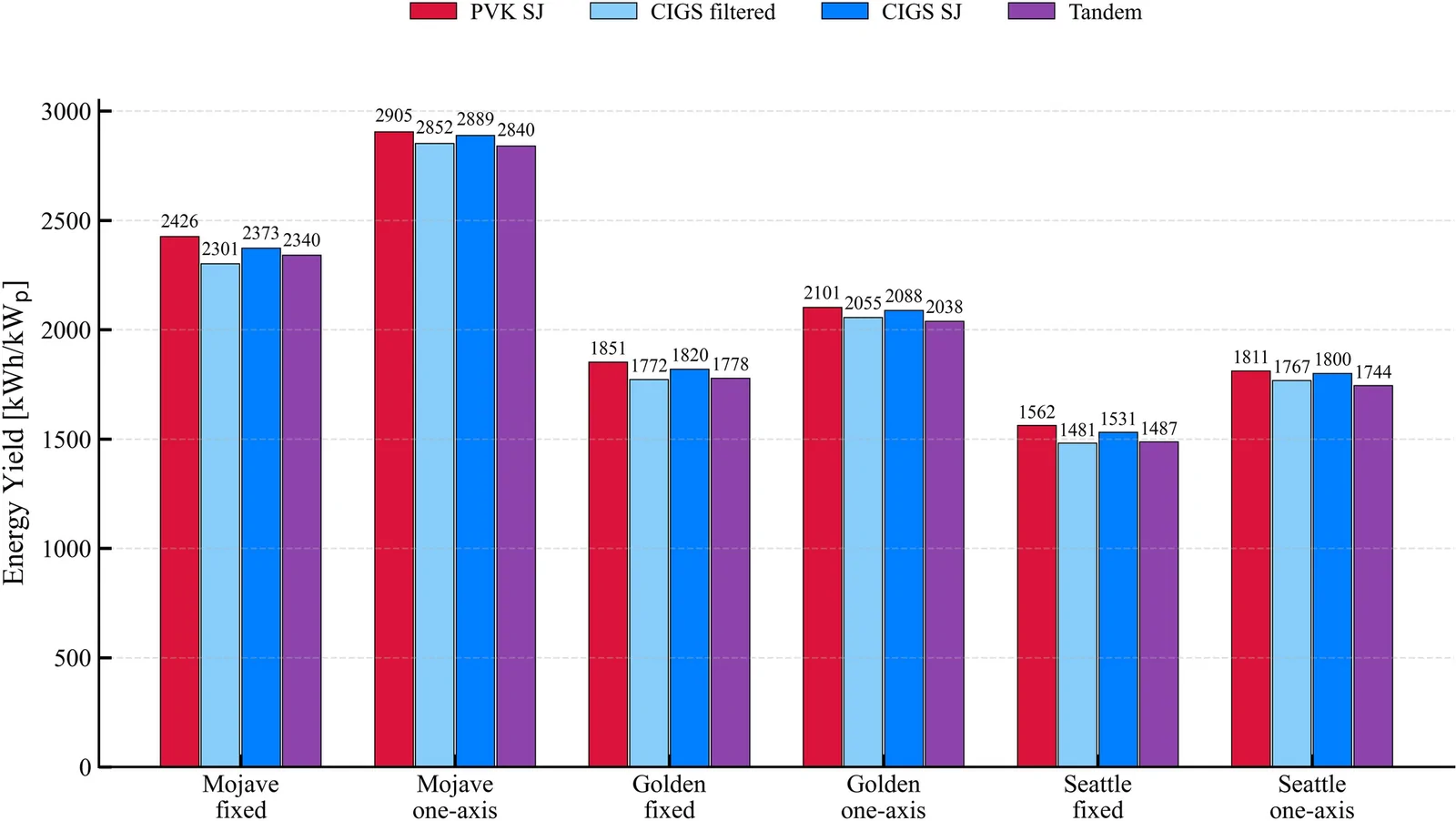
Energy Yield (EY, kWh/kW_p_) for Mojave, Golden, and Seattle under fixed and one-axis tracking, Case 3. Comparison among perovskite SJ, CIGS SJ, CIGS-filtered (bottom sub-cell), and the PVK/CIGS tandem

Moreover, the annual energy yield normalized to the STC peak power (EY, kWh/kW_p_) was used to quantify field performance. Using Case 3 as an example (Fig. 6), both stand-alone cells exhibit higher values across all locations, while the tandem shows a marginal ~ 2%–5% reduction, reflecting current-matching limitations under spectro-thermal variability.

Overall, the stepwise optimization pathway shows that targeted mitigation of electrical and optical losses yields substantial gains in both efficiency and energy yield, strengthening the competitiveness of perovskite/CIGS tandems across diverse deployment scenarios.

### Bandgap Engineering

Tuning the perovskite bandgap (*E*_*g*_) is a well-established strategy to enhance tandem-cell performance. Modelling studies indicate that values in the 1.65–1.75 eV range maximize annual energy yield by combining higher open-circuit voltage with sufficient near-infrared transmission to the CIGS bottom cell [22]. Guided by these insights, we explored a higher-bandgap configuration by increasing ${E}_{g}$ from the baseline 1.63–1.68 eV, while keeping the CIGS absorber unchanged (i.e. 1.05 eV). To preserve current matching, the perovskite thickness was increased from 500 nm (previous case, 1.63 eV) to 800 nm (new case, 1.68 eV), which we identified as optimal for the new stack; otherwise, the CIGS bottom cell would deliver excess current, exacerbating the current mismatch.

From a theoretical perspective, widening the bandgap provides two complementary advantages: (i) a higher attainable *V*_oc_, and (ii) improved spectral allocation by transmitting more near-infrared photons to the CIGS sub-cell, thereby enhancing its photocurrent. These benefits, however, can only be realized if material quality is preserved, since wide-bandgap perovskites are particularly prone to phase segregation and non-radiative recombination.

**Fig. 7:**
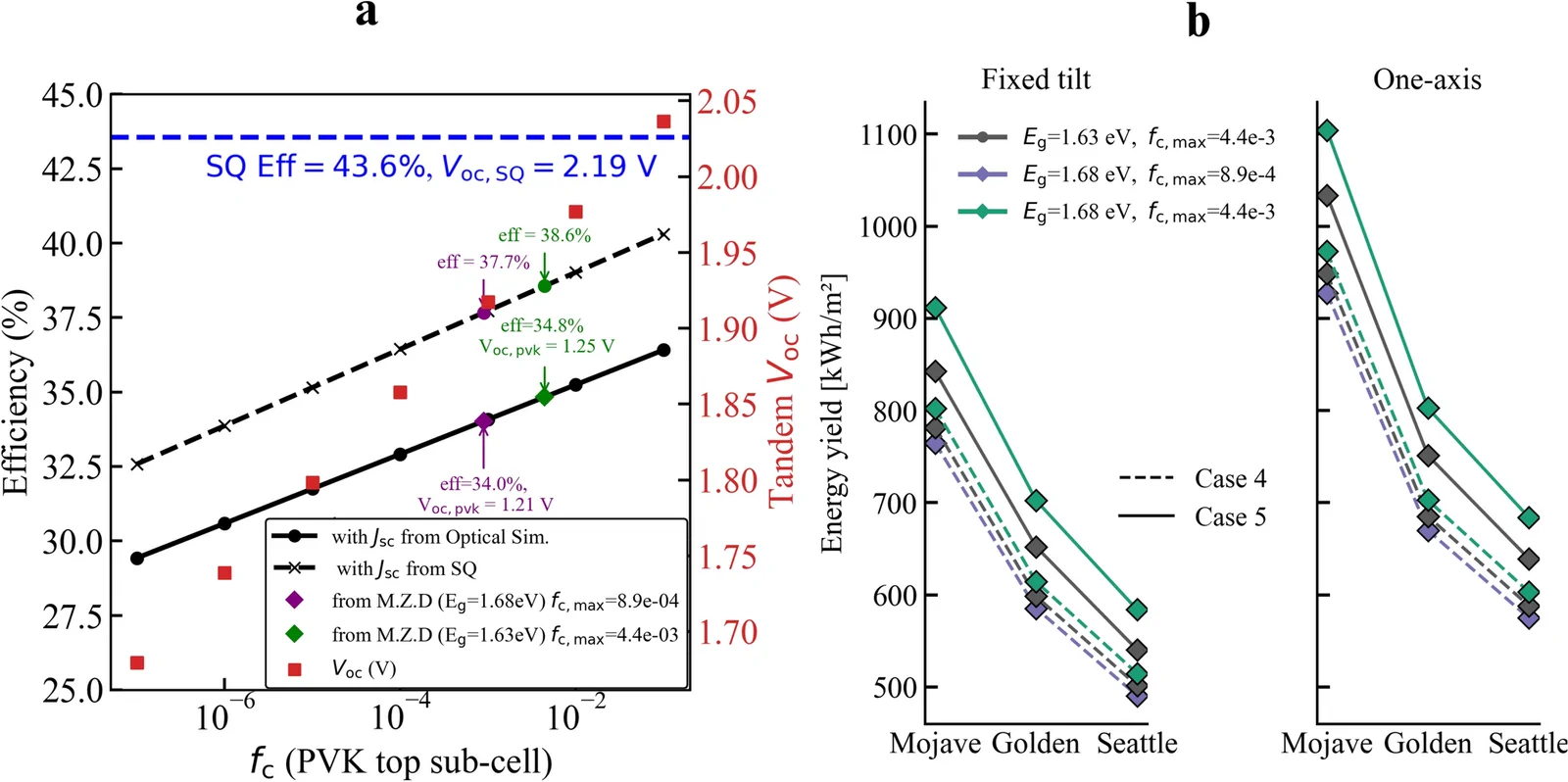
**a** Simulated tandem efficiency and *V*_oc_ as a function of the perovskite top-cell non-radiative recombination factor (*f*_*c*_). Solid lines correspond to the optical TMM–RT case, while dotted lines indicate the ideal absorption limit. **b** Annual energy yield for both bandgaps across three climatic conditions under fixed-tilt and single-axis tracking configurations. M.Z.D. denotes the MaterialZone Database

Figure 7a shows the efficiency and *V*_oc_ of the tandem cell as a function of the pvk top sub-cell non-radiative recombination factor *f*_*c*_, comparing two modelling approaches: (i) physics-based optical simulation of the full stack combined with the M-SQ model, accounting for *f*_*c*_ (Case 4, Sect. 3.2), and (ii) the M-SQ model with ideal optics (Case 5, Sect. 3.2). This provides a direct evaluation of performance as a function of material quality and establishes a reference for comparison with the baseline 1.63 eV configuration, which, as reported in Sect. 3.2, reaches 33.3% (Case 4) and 35.5% (Case 5). To ensure consistency, the 1.68 eV configuration was then evaluated using both its best MaterialZone Database (M.Z.D) value ($f_{c,\max }^{1.68eV} \approx 9 \times 10^{ - 4}$, purple point in Fig. 7a) and the best available value reported for ${E}_{\mathrm{g}}$ = 1.63 eV ($f_{c,\max }^{1.63eV} \approx 4 \times 10^{ - 3}$ green point in Fig. 7a).

Although wide-bandgap perovskites are more prone to non-radiative losses, which limit the attainable *V*_oc_, the 1.68 eV device still achieves 34%–35% efficiency (see Fig. 7a) under the two *f*_c_ assumptions, and approaches 38% when optical improvements are included, thus outperforming the 1.63 eV reference in all cases.

These efficiency gains translate directly into an improved annual energy yield (Fig. 7b). Across all simulated climates (Mojave, Golden, Seattle), the higher-bandgap configuration systematically outperforms the 1.63 eV when high material quality is assumed. However, when the less favourable ${f}_{c\text{, max}}^{\mathrm{1.68eV}}$ is applied (Case 4), the energy yield falls below that of the 1.63 eV reference, underscoring that the benefits of bandgap widening are strongly contingent on material quality. The absolute advantage is greatest under high-irradiance conditions with tracking, while relative improvements are still evident in diffuse-light environments such as Seattle, confirming that bandgap widening provides a robust pathway to higher energy yield, provided non-radiative losses are adequately controlled.

In summary, modest increases in perovskite bandgap can yield tangible improvements in both efficiency and annual energy yield, but only if high material quality is preserved at wider *E*_*g*_. Since a higher *E*_*g*_ reduces top-cell absorption, the perovskite thickness must be increased (from ~ 500 to ~ 800 nm in our case) to sustain current matching. Meeting this requirement will demand progress in stability and defect mitigation, which remain critical challenges for wide-bandgap perovskites.

## Conclusions

This work presents a comprehensive numerical analysis of perovskite/CIGS tandem solar cells, combining state-of-the-art experiments with calibrated optoelectronic modelling. Starting from a certified 24.6% PCE device from HZB as reference, we identified the dominant loss mechanisms by explicitly incorporating measured CIGS grading and surface roughness, and validated our simulations against experimental *EQE* and J-V data. A key aspect of this work is the integration of a modified Shockley–Queisser framework that incorporates empirical non-radiative recombination factors derived from the MaterialZone database.

The stepwise mitigation pathway demonstrates that suppressing recombination at the CdS/CIGSe interface and controlling CIGSe surface morphology can lift the potential efficiency of perovskite/CIGS tandems from ~ 26% to above 31%. Building on these insights, our analysis indicates that reaching efficiencies beyond 35% under AM1.5G illumination in principle requires state-of-the-art material quality combined with advanced transport-layer interface engineering and targeted optical optimization to minimize residual parasitic losses. Annual energy-yield simulations confirm that these gains are robust across diverse climates, with relative improvements particularly relevant under spectrally variable and low-irradiance conditions. Bandgap engineering of the perovskite top cell, by increasing *E*_*g*_ from 1.63 to 1.68 eV, can deliver efficiency gains of up to 3 percentage points and consistent improvements in annual energy yield. However, these benefits are strongly dependent on maintaining high material quality, as non-radiative losses can otherwise offset the advantages of a wider bandgap. In addition, increasing the top-cell thickness is required to sustain current matching, highlighting the need for parallel progress in stability and defect passivation to fully exploit the potential of wide-bandgap perovskites when combined with advanced light-management strategies.

Overall, our results draw a roadmap of performance improvements that are both technologically realistic and anchored in empirical material-quality data. Importantly, adapting the Shockley–Queisser framework with database-derived recombination factors shifts the analysis from idealized limits to realistic performance benchmarks based on fabricated cells. This data-oriented approach not only refines practical efficiency predictions but also provides a rapid and transferable tool for identifying optimal bandgaps and design priorities, complementing detailed physical modelling.

Looking ahead, efficiencies beyond 35% point to the considerable untapped potential of perovskite/CIGS tandem solar cells as a thin-film alternative to two-terminal (2T) perovskite/silicon devices. Further gains could come from three-terminal [57, 58] or four-terminal architectures [59, 60], which relax current-matching constraints and open additional performance pathways. Although, these architectures are still less mature than 2T tandem solar cells, and only marginally explored for perovskite/CIGS, they represent a promising direction for future research. At the same time, recent studies have demonstrated that thermo-mechanical stress at the perovskite/CIGS interconnection [10], originating from thermal expansion mismatch, residual processing-induced strain, and contact morphology, can critically impair operational stability under elevated temperature operation and thermal cycling by promoting defect formation, ion migration, and interfacial degradation [61, 62]. These findings highlight the importance of interfacial stress management through both material-level strategies, such as contact and interface engineering aimed at reducing residual tensile stress and strain localization [10, 61, 63], and system-level design choices that mitigate thermo-mechanical constraints at the device and module level [23]. With continued progress in wide-bandgap perovskite interface control and stability, together with appropriate mechanical and system-level design, perovskite/CIGS tandem solar cells could realistically approach 40% PCE, reinforcing their promise as lightweight, flexible, and versatile photovoltaic technologies for both terrestrial and space applications.

## Supplementary Information

Below is the link to the electronic supplementary material.

Supplementary file1 (DOCX 3093 kb)
