# Supplementary material for: Assessment and Optimization of 2T Perovskite/CIGS Tandems via Data-Driven and Optoelectronic Modelling
Source: Nanomicro Lett. 2026 Mar 31;18:312. doi: 10.1007/s40820-026-02141-8 (PMC13038754; doi:10.1007/s40820-026-02141-8)
Supplement: Supplementary file 1 — Supplementary file1 (DOCX 3093 kb) [file 40820_2026_2141_MOESM1_ESM.docx]

Supporting Information for

**Assessment and Optimization of 2T Perovskite/CIGS Tandems via Data-Driven and Optoelectronic Modelling**

Gemma Giliberti^1^, Guillermo Farias-Basulto^2^, Klaus Jäger^3,4^, Thede Mehlhop^2^, Christian A. Kaufmann^2^, and Aldo Di Carlo^5, 1^*

^1^ CHOSE – Centre for Hybrid and Organic Solar Energy, Department of Electronic Engineering, Tor Vergata University of Rome, via del Politecnico 1, 00133, Rome, Italy

^2^ PvcomB/Helmholtz-Zentrum Berlin für Materialien und Energie GmbH, Schwarzschildstr. 3, 12489 Berlin, Germany

^3^ Department Optics for Solar Energy, Helmholtz-Zentrum Berlin für Materialien und Energie GmbH, Albert-Einstein-Str. 16, 12489 Berlin, Germany

^4^ Computational Nano Optics, Zuse Institute Berlin, Takustr. 7, 14195 Berlin, Germany

^5^ CNR-ISM Consiglio Nazionale delle Ricerche – Istituto di Struttura della Materia, via del Fosso del Cavaliere 100, 00133 Rome, Italy

*Corresponding author. E-mail: [aldo.dicarlo@cnr.it](mailto:aldo.dicarlo@cnr.it) (Aldo Di Carlo)

**S1 Modelling Framework**

The simulation workflow adopted in this study integrates optical, electrical, and circuit-level modules to analyse and optimise the performance of monolithic perovskite/CIGS tandem solar cells under both standard and realistic operating conditions. The complete scheme is illustrated in Figure S1 and is structured around two complementary approaches:

- Case A: full optoelectronic simulations calibrated against experimental *J–V* and *EQE* data;
- Case B: semi-analytical performance modelling based on external absorption spectra and empirical voltage parameters.

The process begins with the definition of the device stack, reproducing the experimental architecture in terms of layer sequence and material properties. Optical simulations are carried out using either the Net Radiation Model (GenPro4) [S1] or equivalently the Transfer Matrix Model (RayFlare) [S2], combined with ray tracing to capture surface roughness and both coherent and incoherent effects [S3]. For the CIGS absorber, spatially varying complex refractive index data ($n$*,* $k$) are obtained through a custom interpolation routine (Section S1.2), which adapt reference datasets to the measured Ga/(Ga+In) bandgap grading profile. These simulations yield either the wavelength- and depth-resolved generation profile $G(\lambda, z)$ (Case A) or the absorptance spectrum $A(\lambda)$ (Case B).

In Case A, $G(\lambda, z)$ serves as input to Poisson–drift–diffusion simulations, which resolve carrier generation, transport, and recombination under illumination. The simulated $\mathrm{EQE}(\lambda)$ and $J$–$V$ curves are benchmarked against experimental data for both standalone sub-cells and the complete tandem, ensuring consistency across optical and electrical models.

In Case B, the absorptance spectrum $A(\lambda)$ is passed into a modified Shockley–Queisser (SQ) framework that incorporates a non-radiative recombination factor $f_{c}$[S4]. Values of $f_{c}$ are extracted using an empirical routine based on the MaterialZone database [S5], which compiles device metrics across bandgaps, architectures, and fabrication conditions (see Sec. S1.4).

Both approaches can be extended to predict the annual energy yield (EY) [S6]. The photocurrent densities $J_{\mathrm{ph}}^{\mathrm{top}}$and $J_{\mathrm{ph}}^{\mathrm{bot}}$are obtained by convoluting the generation profiles (Case A) or absorptance spectra (Case B) with solar irradiance data from the NREL Typical Meteorological Year (TMY) database [S7, S8]. Together with diode parameters (ideality factors, series and shunt resistances), these quantities are used to build a SPICE-equivalent tandem model that computes hourly $J$–$V$ curves and maximum power points, $P_{\mathrm{MPP}}$​. The EY is then obtained by integrating $P_{\mathrm{MPP}}\times\Delta t$ over the full year.


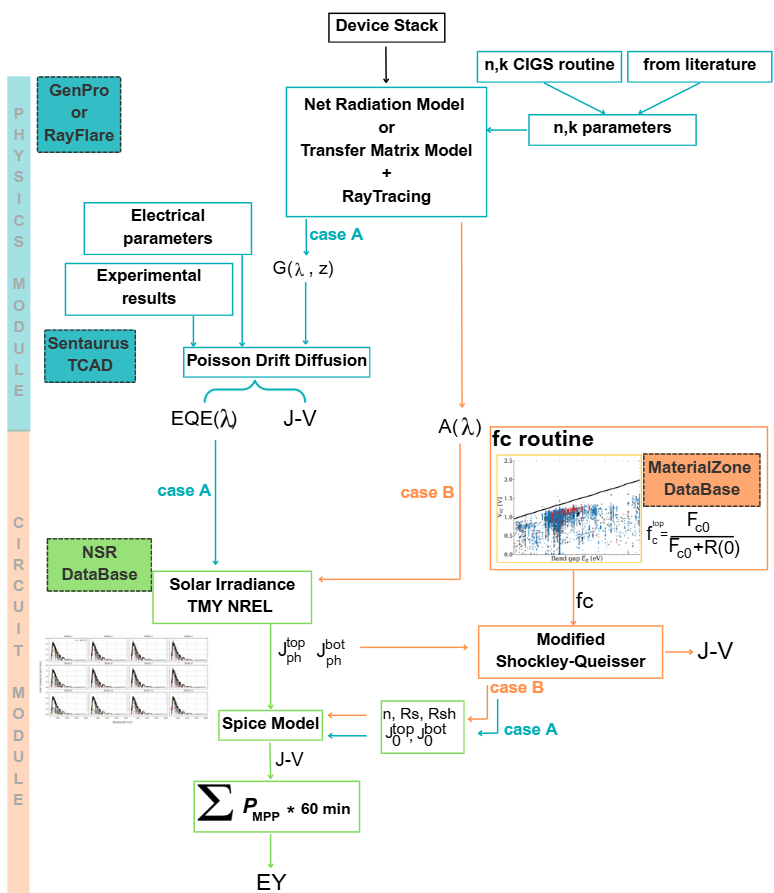


**Fig. S1** Schematic overview of the multiscale simulation workflow employed in this study. Case A (blue) represents physics-based optoelectronic simulations combining optical generation profiles with TCAD drift-diffusion modelling. Case B (orange) corresponds to a semi-empirical approach based on external absorption spectra and non-radiative recombination factors *f*_c_ extracted from the MaterialZone database

**S1.1 Details of Optical Simulations with Genpro4**

Table S1 shows the detailed layer stack used for the optical simulations with GenPro4 [S1], including the sources of the complex refractive index (*n*,*k*) data. In the simulations, light propagation in the perovskite, CIGS and Mo layers was treated incoherently, while it was treated coherently in all other layers. The ~1 nm LiF interlayer and the self-assembled monolayer on top and bottom of the perovskite layer, respectively, were omitted in the simulations. The interfaces between the CIGS bottom and the perovskite top absorbers were assumed to have the roughness of a generic CIGS surface, shown in Fig. S2b. In the optical simulation, scattering at these interfaces was treated with ray tracing. All other interfaces were treated as optically flat.

**Table S1** The layer stack used for the optical simulations

| Material | *reference* | *Thickness (nm)* |
| --- | --- | --- |
| LiF | [S9] | 100 |
| IZO | [S10] | 40 |
| SnO_2_ | [S11] | 20 |
| C_60_ | [S12] | 23 |
| perovskite | [S7] | 500 |
| NiO_x_ | [S13] | 15 |
| AZO | characterized at HZB | 60 |
| i-ZnO | [S1] | 40 |
| CdS | [S1] | 50 |
| CIGS | *See section S1.2* | 2200 |
| Mo | [S1] | 800 |

**S1.2 Optics: Complex Refractive Index Of CIGS**

Modelling the CIGS bottom sub-cell requires a detailed description of its depth-dependent refractive index $n$ and extinction coefficient $k$, which vary with the compositional grading of the absorber.


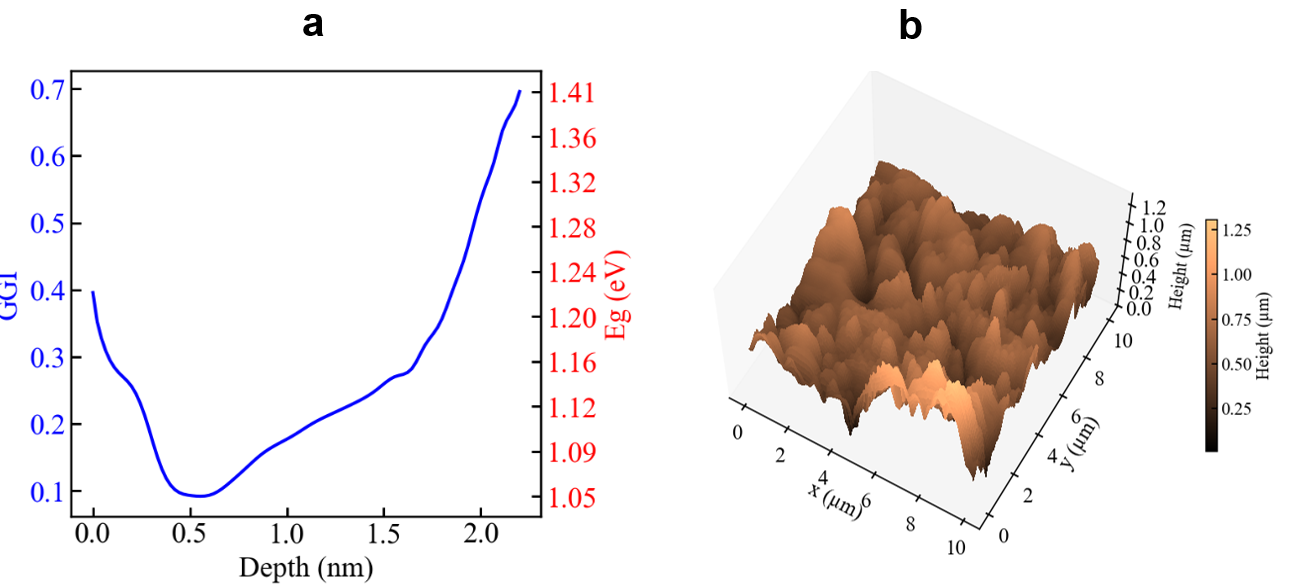


**Fig. S2** **a** Depth-dependent GGI profile of the CIGS layer. The GGI content is plotted on the left axis, with the corresponding bandgap shown on the right axis, calculated via Eq.S1. **b** 3D surface reconstruction of the CIGS bottom sub-cell (HZB), derived from AFM measurements of the CIGS sub-cell

Since full experimental datasets covering the entire bandgap range are not available, we generated a consistent set of optical constants to reflect the local variations in Ga/(Ga+In) ratio (GGI), derived from our compositional profiling (Fig. S2a). The experimental GGI depth profile [S14], as determined by GDOES in-depth profiling, reproduced in Figure S2a, is converted into a corresponding bandgap profile using the semi-empirical expression of Eq. S1 [S15]:

$$E_{g}\left( x \right)=1.01+0.626x-0.167\left( x-x^{2} \right) ( S1)$$

where $x$ is the local GGI content. The resulting bandgap spans from approximately 1.05 eV near the front interface to ~1.41 eV at the back contact, following a characteristic “V-shaped” grading with a minimum located at approximately 500 nm depth. To capture these effects, the CIGS layer was discretized into a series of thin sub-layers. Each sub-layer was assigned optical constants corresponding to its local bandgap value. However, since experimental $(n,k)$ data are typically limited to specific compositions, we developed a Python-based interpolation routine to generate a continuous material database. This approach builds on the methodology introduced by Hörantner and Snaith [S7] for perovskite/silicon tandems. Two experimental datasets were selected as optical references: one corresponding to CIGS with GGI = 0.15 ($E_{g} \approx1.18 \mathrm{eV}$) for low bandgaps, and another with GGI = 0.30 ($E_{g} \approx1.32 \mathrm{eV}$) for higher bandgaps. The extinction coefficient $k(\lambda)$ was adjusted for intermediate values by spectrally shifting the original spectrum to match the target bandgap, as shown in Figure S3a. The accuracy of the shift was verified using Tauc analysis (Figure S3b). Once the adjusted$k(\lambda)$ was obtained, the corresponding real part$n(\lambda)$ was computed via Kramers–Kronig transformation using the open-source pykk.py library.

Figure S4 shows the resulting set of $n(\lambda)$ and $k(\lambda)$ spectra for several bandgaps, demonstrating consistent variation across the GGI range. This framework enables accurate reconstruction of the depth-dependent optical properties of the graded CIGS layer, crucial for realistic optical simulation and device modelling. The same approach is used also to get optical data for perovskites with varying bandgaps.

**
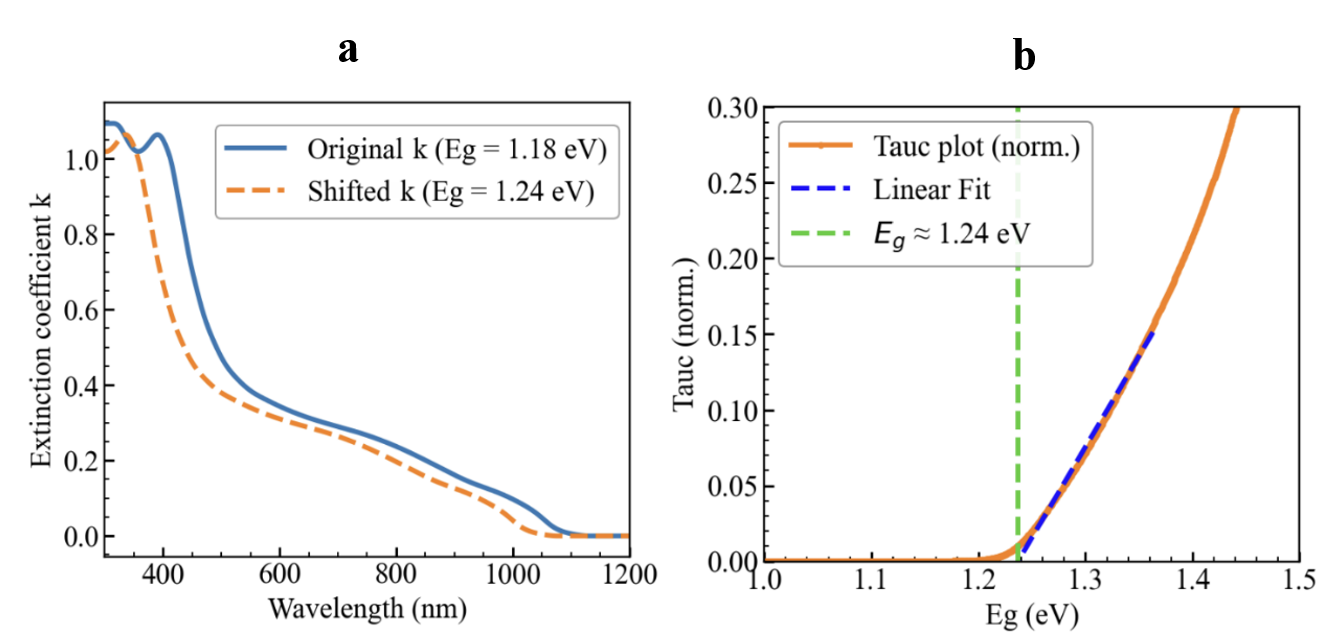
**

**Fig. S3** Generation of CIGS optical constants (n,k) for depth-dependent bandgaps. **a** Spectral shift of the extinction coefficient k(λ) to match a target bandgap. **b** Tauc plot used to extract the corresponding *E*_g_

**
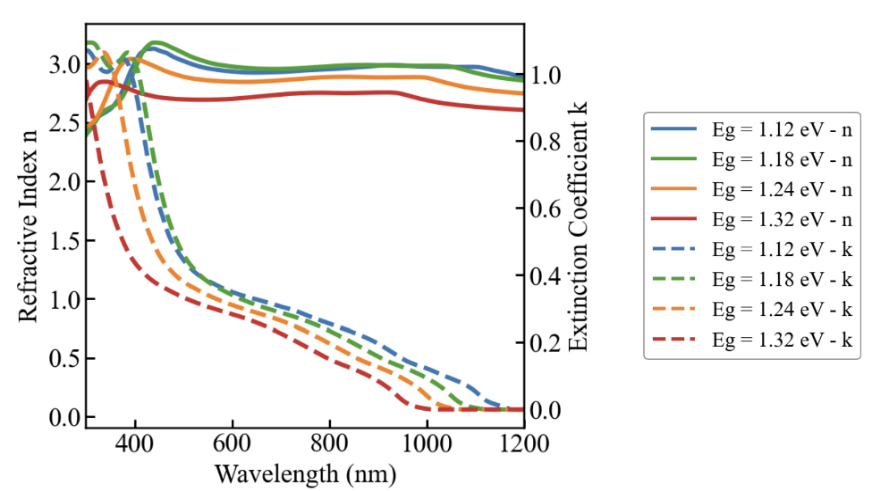
**

**Fig. S4** n(λ) and k(λ) for several Eg values via Kramers–Kronig transformation

**S1.3 Electrical Parameters**

**Table S2** Electrical parameters used for the simulation of the monolithic perovskite/CIGS tandem solar cell. Values include electron affinity (*χ*), bandgap (*E*_g_), doping concentration, carrier mobilities, effective density of states (DOS) for Conduction/Valence band (C/VB). For cigs cell parameters refers to Procel et al. article [S16]

| Parameter | IZO  [S17–S19] | SnO_2_  [S16, S17, S20] | C_60_  [S17, S20] | PVK  [S16, S21] | 2PACz  [S22] | NiO_x_  [S16, S21, S22] | AZO  [S23] |
| --- | --- | --- | --- | --- | --- | --- | --- |
| *E*_g_ [eV] | 3.6 | 4.15 | 2 | 1.63 | 3.6 | 3.84 | 3.4 |
| $\boldsymbol{\chi}$ [eV] | 3.81 | 3.85 | 4 | 4.12 | 1.75 | 1.4 | 4.6 |
| Doping [cm^-3^] | 3.4e20 | 1e18 | 1e18 | 5e14 | 1e18 | 4.63e19 | 2.3e20 [exp.] |
| Mob. e [cm^2^V^-1^s^-1^] | 43.5 | 0.001 | 0.01 | 35 | 0.001 | 0.001 | 15 [exp.] |
| Mob. h [cm^2^V^-1^s^-1^] | 0.001 | 0.25 | 0.01 | 35 | 0.001 | 0.001 | 15 [exp.] |
| CB DOS [cm^-3^] | 1e19 | 4e18 | 2.2e18 | 1e20 | 1e20 | 2.2e18 | 4e18 |
| VB DOS [cm^-3^] | 1e19 | 1e18 | 1.8e18 | 1e19 | 1e19 | 1.8e19 | 1e18 |
| Permittivity | 10 | 10 | 5 | 6.5 | 11.9 | 11.9 | 10 |

**Table S3** Defect density of tandem cell

| Materials | Defect Density [cm^-3^eV⁻¹] | Energy Level | Capture Cross Section [cm²s^-1^] | Type |  |
| --- | --- | --- | --- | --- | --- |
| C₆₀ / Perovskite | 10^12^ | Mid-gap | 1 × 10⁻^17^ (e/h) | Donor |  |
| Perovskite | | 1.5 × 10^14^ | Mid-gap | 1 × 10⁻^16^ (e/h) | Donor |
| Perovskite / SAM | | 10^10^ | Mid-gap | 1 × 10⁻^17^ (e/h) | Donor |
| CdS/CIGS | | 2 × 10^15^ | 0.2 from CB | 5 × 10⁻^16^ (e) 1 × 10⁻^18^ (h) | Acceptor |
| CdS/CIGS | | 2 × 10^15^ | 0.2 from VB | 1 × 10⁻^18^ (e) 5 × 10⁻^16^ (h) | Donor |

**S1.4 Experimental Results**

**Experimental Methods**

Monolithic two-terminal perovskite/CIGS tandem solar cells were fabricated by integrating a co-evaporated CIGS bottom cell with a solution-processed wide-bandgap perovskite top cell. The CIGS absorber (∼2.2–2.4 µm) was grown on Mo-coated soda-lime glass using a three-stage co-evaporation process followed by an RbF post-deposition treatment. Bandgap reduction was achieved by lowering the substrate temperature during the second and third stages (530 → 490 °C), thereby modifying the Ga depth profile. A CdS buffer layer was deposited by chemical bath deposition, followed by sputtered i-ZnO/Al:ZnO as the electron-transport/window stack.

For the interconnection to the top cell, a nanoparticulate NiO_x_ layer was spin-coated after UV–ozone treatment of the CIGS surface, followed by deposition of a 2PACz self-assembled monolayer. The perovskite top cell employed a triple-cation composition with a reduced bandgap (~1.63 eV), deposited by spin-coating using an antisolvent method and annealed at 100 °C. A LiF passivation layer and a C_60_ electron-transport layer were thermally evaporated. The window/contact stack consisted of ALD SnO_x_, sputtered IZO with reduced thickness (40 nm), and an evaporated Ag top electrode, defining an active area of 1.105 cm². An additional LiF antireflection coating was applied. Further experimental details are reported in [S14].

**Results**


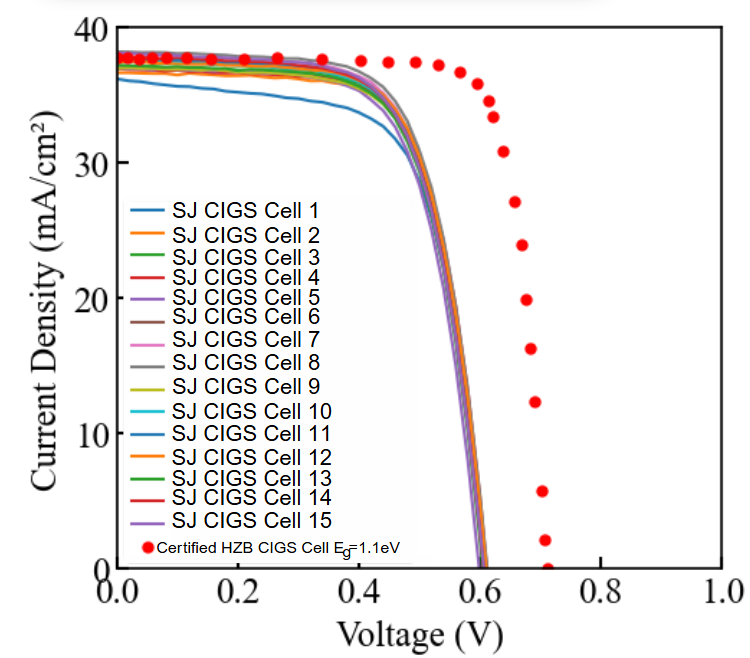

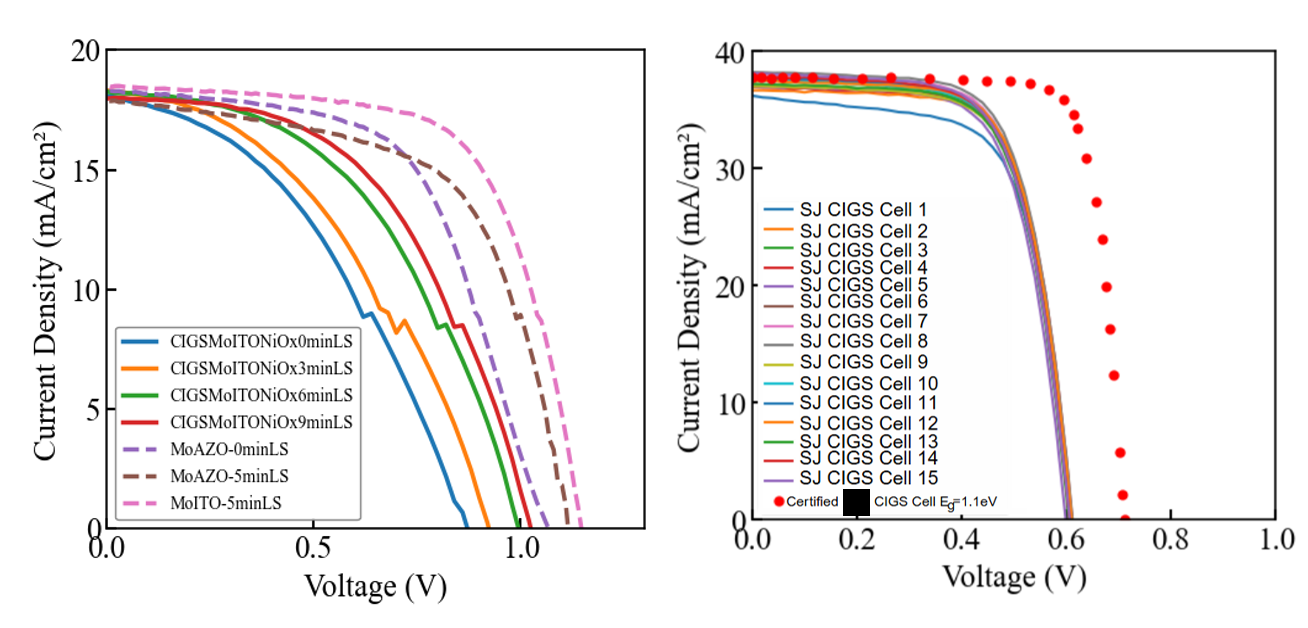


**Fig. S5** Left: Current density–voltage (J–V) characteristics of perovskite top cells fabricated on rough (CIGS/Mo/ITO/NiO_x_) and flat (Mo/AZO, Mo/ITO) bottom electrodes with varying LS durations (0–9 min). The Mo/ITO-5 min LS device shows the best performance among the top cells. Right: J–V curves of 15 reference CIGS bottom cells (*E*_g_ = 1.05eV) extracted from the same sample batch, compared with the certified HZB CIGS cell (red dots) having an effective bandgap of 1.1 eV

**Table S4** Photovoltaic parameters of experimental CIGS devices. Short-circuit current density *J*_sc_, Open-circuit voltage *V*_oc_, Fill-Factor *FF*, Series Resistance *R*_s_, Shunt Resistance *R*_sh_, Efficiency *Eff*

| Spectrum | Type | $\boldsymbol{J}_{\mathbf{sc}}$  [mA/cm²] | $\boldsymbol{V}_{\mathbf{oc}}$ [mV] | *FF* [%] | *Eff.*  [%] | $\boldsymbol{R}_{\mathbf{sh}}$ [Ohm·cm²] | $\boldsymbol{R}_{\mathbf{s}}$ [Ohm·cm²] |
| --- | --- | --- | --- | --- | --- | --- | --- |
| AM1.5 | Best | 37.74 | 609.3 | 68.13 | 15.67 | 759 | 2.23 |
| AM1.5 | Median | 37.38 | 608.2 | 67.97 | 15.48 | 784 | 2.21 |

**Table S5** Photovoltaic parameters of experimental PVK devices. Short-circuit current density *J*_sc_, Open-circuit voltage *V*_oc_, Fill-Factor *FF*, Series Resistance R_s_, Shunt Resistance R_sh_, Efficiency *Eff*

| Meas. | Sub. | $\boldsymbol{J}_{\mathbf{sc}}$ [mA/cm²] | $\boldsymbol{V}_{\mathbf{oc}}$ [V] | *FF* [%] | *Eff.* [%] | $\boldsymbol{R}_{\mathbf{s}}$ [Ohm·cm²] | $\boldsymbol{R}_{\mathbf{sh}}$  [Ohm·cm²] |
| --- | --- | --- | --- | --- | --- | --- | --- |
| 9mLS_rev | CIGS/Mo/ITO/NiO_x_ | 18.30 | 1.03 | 50.90 | 9.4 | 21 | 1354 |
| 5mLS_rev | Mo/ITO | 18.49 | 1.15 | 65.17 | 13.82 | 9.01 | 961.70 |
| 5mLS_rev | Mo/AZO | 17.93 | 1.12 | 60.21 | 12.03 | 7.53 | 455.09 |


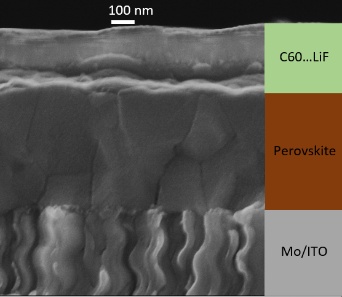

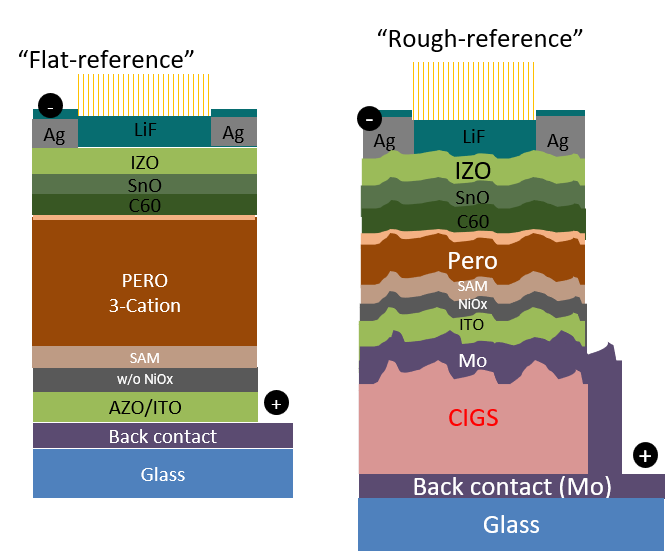

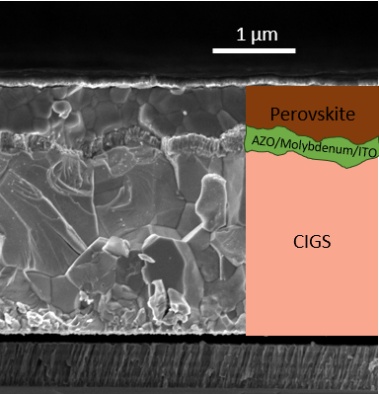


**Fig. S6** Cross-sectional schematics of the “Flat-reference” and “Rough-reference” stand-alone perovskite devices. The flat stack (left) is deposited on a smooth AZO/ITO substrate, while the rough stack (right) replicates the morphology of the CIGS bottom cell, enabling assessment of roughness-induced interfacial effects on perovskite performance

**
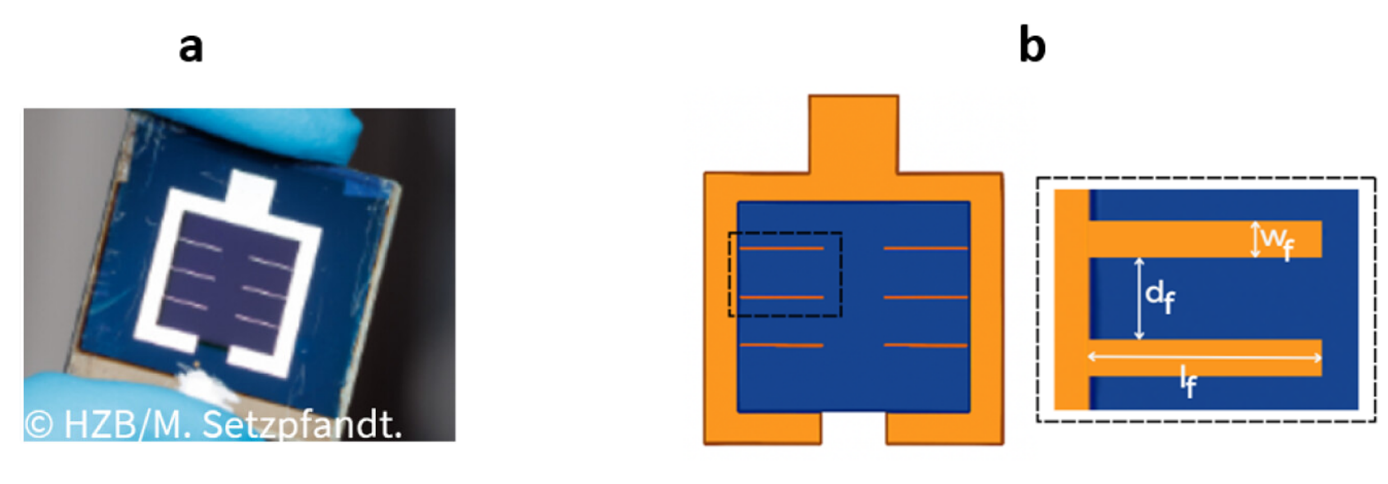
**

**Fig. S7 a** Photograph of the fabricated small-area perovskite/CIGS tandem solar cell. **b** Schematic of the front metal grid layout used in the experimental device. Ag fingers are deposited on top of the IZO front electrode and correspond to the optimised design adopted for the ~1 cm² reference cell. The finger width (w_f_), finger length (l_f_) and pitch (d_f_) are, 30 µm, 4 mm and 2.6 mm, respectively, for an IZO thickness of 40 nm (sheet resistance ∼ 120 Ω/□), providing a balanced trade-off between optical shading and lateral series resistance [S14, S24]

**S1.5 *Recombination Factor* Routine**

The theoretical efficiency limits of photovoltaic devices are typically evaluated using the Shockley–Queisser (SQ) model [S4] under the assumption of purely radiative recombination. However, non-radiative losses can be accounted for by introducing a recombination factor *f*_c_​. Since, to the best of our knowledge, it is uncommon in the literature to recompute these limits using experimental open-circuit voltages from external datasets, we briefly summarize here the methodology and equations employed to extract the *f*_c_ ​ parameter. This approach is based on the MaterialZone Database [S5], which represents a core resource in this work.

$$f_{c}=\frac{J_{o}^{\mathrm{rad}}}{J_{o}^{\mathrm{rad}}+J_{o}^{non-rad}}=\frac{J_{o}^{\mathrm{rad}}}{J_{o}^{\mathrm{tot}}} (S2)$$

Under the assumptions of ideal diode behavior and negligible series resistance, the diode equation at open-circuit ($J = 0$) reads:

$$J_{\mathrm{sc}}=J_{o}*\exp\left( \frac{V_{\mathrm{oc}}}{V_{t}} \right) \to J_{o}=J_{\mathrm{sc}}*\exp\left( -\frac{V_{\mathrm{oc}}}{V_{t}} \right) (S3)$$

By applying this to both the radiative limit and the experimental case:

$$J_{o}^{\mathrm{rad}}= J_{\mathrm{sc}}\times\exp\left( -\frac{V_{\mathrm{oc}}^{\mathrm{rad}}}{V_{t}} \right) (S4)$$

$$J_{o}^{\mathrm{tot}}= J_{\mathrm{sc}}\times\exp\left( -\frac{V_{\mathrm{oc}}^{\exp}}{V_{t}} \right) (S5)$$

Taking the ratio gives:

$$f_{c}=\frac{J_{o}^{\mathrm{rad}}}{J_{o}^{\mathrm{tot}}}= \exp\left( \frac{V_{\mathrm{oc}}^{\exp}-V_{\mathrm{oc}}^{\mathrm{rad}}}{V_{t}} \right) (S6)$$

where:

- $V_{\mathrm{oc}}^{\mathrm{rad}}$is computed from the radiative Shockley–Queisser limit using the classical formalism
- $V_{\mathrm{oc}}^{\exp}$ is extracted from the MaterialZone database, based on the perovskite bandgap and technology
- $V_{t}=\frac{kT}{q}$ where $k$ is the Boltzmann constant, $q$ is the elementary charge, and $T = 300 K$.

This method allows us to consistently parameterize empirical non-radiative recombination within the SQ framework by substituting the ideal saturation current:

$$J_{o}^{\mathrm{rad}}\to\frac{J_{o}^{\mathrm{rad}}}{f_{c}} (S7)$$

**
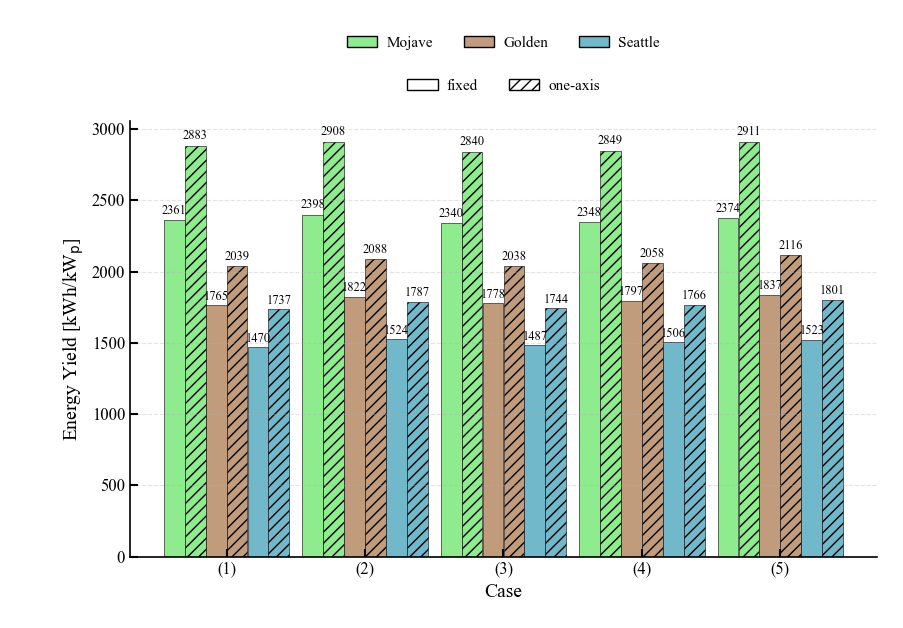
S1.6 Energy Yield**


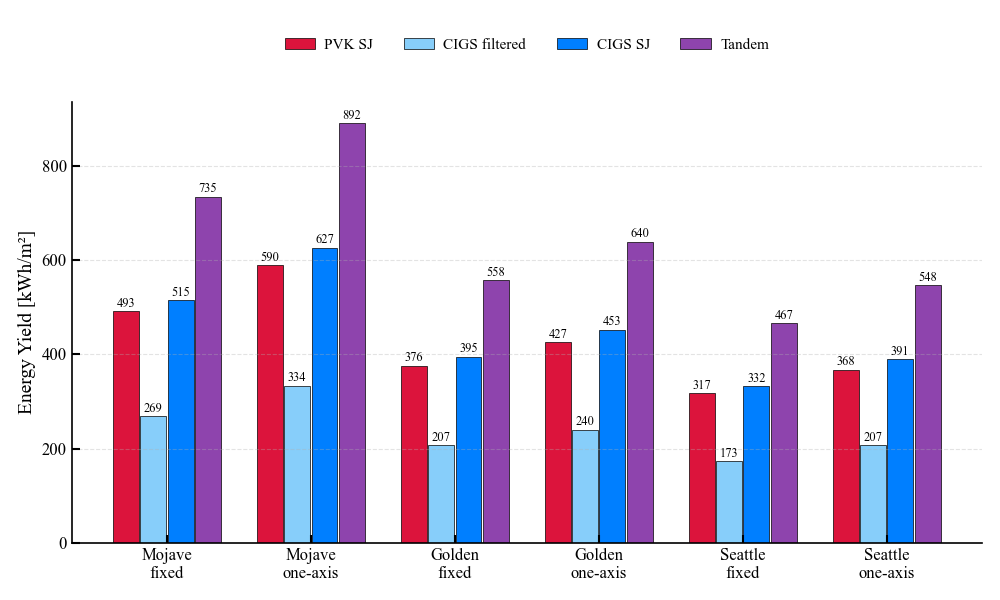
**Fig. S8** Capacity factor for the five step-cases in Mojave, Golden, and Seattle, under fixed-tilt and one-axis tracking configurations

**Fig. S9** Case 3) Annual energy yield (EY) for Mojave, Golden, and Seattle under fixed-tilt and one-axis tracking: comparison between single-junction (SJ) baselines and the tandem architecture

**Supplementary References**

1. R. Santbergen, T. Meguro, T. Suezaki, G. Koizumi, K. Yamamoto et al., GenPro4 optical model for solar cell simulation and its application to multijunction solar cells. IEEE J. Photovolt. **7**(3), 919–926 (2017). <https://doi.org/10.1109/JPHOTOV.2017.2669640>
2. P. Pearce, N. Ekins-Daukes, Open-source integrated optical modelling with Ra*_y_*Flare. 2019 IEEE 46th Photovoltaic Specialists Conference (PVSC). June 16-21, 2019. Chicago, IL, USA. IEEE, (2019).: 2627-2633. <https://doi.org/10.1109/pvsc40753.2019.8980868>
3. G. Giliberti, A. Fronteddu, E. Magliano, F. Matteocci, F. Di Giacomo et al., Development of middle-contact three-terminal perovskite/silicon tandems. Physics, Simulation, and Photonic Engineering of Photovoltaic Devices XIV. January 25-31, 2025. San Francisco, USA. SPIE, (2025).: 14. <https://doi.org/10.1117/12.3040483>
4. W. Shockley, H.J. Queisser, Detailed balance limit of efficiency of*p-n*Junction solar cells. J. Appl. Phys. **32**(3), 510–519 (1961). <https://doi.org/10.1063/1.1736034>
5. T.J. Jacobsson, A. Hultqvist, A. García-Fernández, A. Anand, A. Al-Ashouri et al., An open-access database and analysis tool for perovskite solar cells based on the FAIR data principles. Nat. Energy **7**(1), 107–115 (2022). <https://doi.org/10.1038/s41560-021-00941-3>
6. M. Langenhorst, B. Sautter, R. Schmager, J. Lehr, E. Ahlswede et al., Energy yield of all thin-film perovskite/CIGS tandem solar modules. Prog. Photovolt. Res. Appl. **27**(4), 290–298 (2019). <https://doi.org/10.1002/pip.3091>
7. M.T. Hörantner, H.J. Snaith, Predicting and optimising the energy yield of perovskite-on-silicon tandem solar cells under real world conditions. Energy Environ. Sci. **10**(9), 1983–1993 (2017). <https://doi.org/10.1039/c7ee01232b>
8. National Renewable Energy Laboratory, National solar radiation database (nsrdb). (2017). <https://nsrdb.nrel.gov/>
9. H.H. Li, Refractive index of alkali halides and its wavelength and temperature derivatives. J. Phys. Chem. Ref. Data **5**(2), 329–528 (1976). <https://doi.org/10.1063/1.555536>
10. J. Werner, G. Dubuis, A. Walter, P. Löper, S.-J. Moon et al., Sputtered rear electrode with broadband transparency for perovskite solar cells. Sol. Energy Mater. Sol. Cells **141**, 407–413 (2015). <https://doi.org/10.1016/j.solmat.2015.06.024>
11. M. Jošt, E. Köhnen, A.B. Morales-Vilches, B. Lipovšek, K. Jäger et al., Textured interfaces in monolithic perovskite/silicon tandem solar cells: advanced light management for improved efficiency and energy yield. Energy Environ. Sci. **11**(12), 3511–3523 (2018). <https://doi.org/10.1039/c8ee02469c>
12. D. Menzel, A. Al-Ashouri, A. Tejada, I. Levine, J.A. Guerra et al., Field effect passivation in perovskite solar cells by a LiF interlayer. Adv. Energy Mater. **12**(30), 2201109 (2022). <https://doi.org/10.1002/aenm.202201109>
13. H.L. Lu, G. Scarel, M. Alia, M. Fanciulli, S.-J. Ding et al., Spectroscopic ellipsometry study of thin NiO films grown on Si (100) by atomic layer deposition. Appl. Phys. Lett. **92**(22), 222907 (2008). <https://doi.org/10.1063/1.2938697>
14. G. Farias-Basulto, T. Mehlhop, N.J. Otto, T. Bertram, K. Jäger et al., Improving perovskite/CIGS tandem solar cells for higher power conversion efficiency through light management and bandgap engineering. ACS Appl. Mater. Interfaces **17**(40), 56250–56255 (2025). <https://doi.org/10.1021/acsami.5c15458>
15. C. Frisk, C. Platzer-Björkman, J. Olsson, P. Szaniawski, J.T. Wätjen et al., Optimizing Ga-profiles for highly efficient Cu(In, Ga)Se_2_ thin film solar cells in simple and complex defect models. J. Phys. D: Appl. Phys. **47**(48), 485104 (2014). <https://doi.org/10.1088/0022-3727/47/48/485104>
16. P. Procel, J. Knobbe, N. Rezaei, V. Zardetto, N. Phung et al., Opto-electrical modelling and roadmap for 2T monolithic Perovskite/CIGS tandem solar cells. Sol. Energy Mater. Sol. Cells **274**, 112975 (2024). <https://doi.org/10.1016/j.solmat.2024.112975>
17. A. Al-Ashouri, E. Köhnen, B. Li, A. Magomedov, H. Hempel et al., Monolithic perovskite/silicon tandem solar cell with >29% efficiency by enhanced hole extraction. Science **370**(6522), 1300–1309 (2020). <https://doi.org/10.1126/science.abd4016>
18. Y. Jin, H. Feng, Z. Fang, H. Zhang, L. Yang et al., Efficient and stable monolithic perovskite/silicon tandem solar cells enabled by contact-resistance-tunable indium tin oxide interlayer. Adv. Mater. **36**(35), e2404010 (2024). <https://doi.org/10.1002/adma.202404010>
19. N. Shrivastav, J. Madan, R. Pandey, A.E. Shalan, Investigations aimed at producing 33% efficient perovskite–silicon tandem solar cells through device simulations. RSC Adv. **11**(59), 37366–37374 (2021). <https://doi.org/10.1039/D1RA06250F>
20. H.M. Pham, S.D.H. Naqvi, H. Tran, H. Van Tran, J. Delda et al., Effects of the electrical properties of SnO_2_ and C60 on the carrier transport characteristics of p-i-n-structured semitransparent perovskite solar cells. Nanomaterials **13**(24), 3091 (2023). <https://doi.org/10.3390/nano13243091>
21. A. Al-Ashouri, A. Magomedov, M. Roß, M. Jošt, M. Talaikis et al., Conformal monolayer contacts with lossless interfaces for perovskite single junction and monolithic tandem solar cells. Energy Environ. Sci. **12**(11), 3356–3369 (2019). <https://doi.org/10.1039/c9ee02268f>
22. D. Wang, Z. Liu, Y. Qiao, Z. Jiang, P. Zhu et al., Rigid molecules anchoring on NiO*_x_* enable >26% efficiency perovskite solar cells. Joule **9**(3), 101815 (2025). <https://doi.org/10.1016/j.joule.2024.101815>
23. I. Kafedjiska, I. Levine, A. Musiienko, N. Maticiuc, T. Bertram et al., Advanced characterization and optimization of NiOx: Cu-SAM hole-transporting bi-layer for 23.4% efficient monolithic Cu(In, Ga)Se_2_-perovskite tandem solar cells. Adv. Funct. Mater. **33**(34), 2302924 (2023). <https://doi.org/10.1002/adfm.202302924>
24. S. Mariotti, E. Köhnen, F. Scheler, K. Sveinbjörnsson, L. Zimmermann et al., Interface engineering for high-performance, triple-halide perovskite-silicon tandem solar cells. Science **381**(6653), 63–69 (2023). <https://doi.org/10.1126/science.adf5872>
